# Floral regulators FLC and SOC1 directly regulate expression of the B3-type transcription factor TARGET OF FLC AND SVP 1 at the Arabidopsis shoot apex via antagonistic chromatin modifications

**DOI:** 10.1371/journal.pgen.1008065

**Published:** 2019-04-04

**Authors:** René Richter, Atsuko Kinoshita, Coral Vincent, Rafael Martinez-Gallegos, He Gao, Annabel D. van Driel, Youbong Hyun, Julieta L. Mateos, George Coupland

**Affiliations:** 1 Department of Plant Developmental Biology, Max-Planck-Institute for Plant Breeding, Cologne, Germany; 2 Fundación Instituto Leloir, IIBBA-CONICET, Buenos Aires, Argentina; National University of Singapore and Temasek Life Sciences Laboratory, SINGAPORE

## Abstract

Integration of environmental and endogenous cues at plant shoot meristems determines the timing of flowering and reproductive development. The MADS box transcription factor FLOWERING LOCUS C (FLC) of *Arabidopsis thaliana* is an important repressor of floral transition, which blocks flowering until plants are exposed to winter cold. However, the target genes of FLC have not been thoroughly described, and our understanding of the mechanisms by which FLC represses transcription of these targets and how this repression is overcome during floral transition is still fragmentary. Here, we identify and characterize *TARGET OF FLC AND SVP1 (TFS1)*, a novel target gene of FLC and its interacting protein SHORT VEGETATIVE PHASE (SVP). *TFS1* encodes a B3-type transcription factor, and we show that *tfs1* mutants are later flowering than wild-type, particularly under short days. FLC and SVP repress *TFS1* transcription leading to deposition of trimethylation of Iysine 27 of histone 3 (H3K27me3) by the Polycomb Repressive Complex 2 at the *TFS1* locus. During floral transition, after downregulation of *FLC* by cold, *TFS1* transcription is promoted by SUPPRESSOR OF OVEREXPRESSION OF CONSTANS1 (SOC1), a MADS box protein encoded by another target of FLC/SVP. SOC1 opposes PRC function at *TFS1* through recruitment of the histone demethylase RELATIVE OF EARLY FLOWERING 6 (REF6) and the SWI/SNF chromatin remodeler ATPase BRAHMA (BRM). This recruitment of BRM is also strictly required for SQUAMOSA PROMOTER BINDING PROTEIN-LIKE 9 (SPL9) binding at *TFS1* to coordinate RNAPII recruitment through the Mediator complex. Thus, we show that antagonistic chromatin modifications mediated by different MADS box transcription factor complexes play a crucial role in defining the temporal and spatial patterns of transcription of genes within a network of interactions downstream of FLC/SVP during floral transition.

## Author summary

The initiation of flowering in plants is exquisitely sensitive to environmental signals, ensuring that reproduction occurs at the appropriate time of year. The sensitivity of these responses depends upon strong repression of flowering under inappropriate conditions. FLOWERING LOCUS C (FLC) and SHORT VEGETATIVE PHASE (SVP) are related transcription factors that act in concert to strongly inhibit flowering in crucifer plants through repressing transcription of their target genes. Many direct FLC/ SVP targets have been identified in genome-wide studies, however few of these genes have been characterized for their roles in regulating flowering time or other aspects of reproductive development. Here, we characterize *TARGET OF FLC AND SVP1* (*TFS1*) as a novel target of FLC and SVP, and demonstrate that *TFS1* contributes to proper flowering-time control. Moreover, we provide a detailed mechanistic view of how *TFS1* transcription is controlled during reproductive development through the repressive activity of FLC/SVP being overcome by the transcriptional activator SUPPRESSOR OF OVEREXPRESSION OF CONSTANS 1. Thus we further elucidate the network of genes repressed by FLC/SVP to block flowering and determine mechanisms by which their repressive activity is overcome during the initiation of flowering.

## Introduction

The transition from vegetative to reproductive development in plants is controlled by a complex transcriptional network that responds both to environmental cues and endogenous hormonal signals [1, 2]. In *Arabidopsis thaliana*, the MADS-box transcription factor FLOWERING LOCUS C (FLC) plays a major role in this network as an inhibitor of floral transition [3, 4]. Transcription of *FLC* is repressed by extended exposure to cold that mimics winter conditions (vernalization) so that flowering can proceed when plants are subsequently exposed to warm in spring. The repression of *FLC* transcription by accumulation of histone modifications in response to cold has been extensively studied [5] and FLC target genes have been described by whole genome chromatin immunoprecipitation (ChIPseq) [6–8]. Nevertheless, our understanding of how FLC influences the transcriptional network that controls floral transition and how it represses expression of its target genes is still fragmentary. Here, we utilize data derived from genome-wide binding studies of FLC and its partner MADS box transcription factor SHORT VEGETATIVE PHASE (SVP) [7, 9–12] to identify a common target gene that we named *TARGET of FLC and SVP 1* (*TFS1*). We show that this gene acts in the network downstream of FLC and other floral regulators, and has an important role on the flanks of the meristem during the early stages of floral development.

FLC binds to several hundred target genes, but only a small subset of these are conserved between species [8]. Genes involved in flowering control are enriched among the conserved targets, and FLC represses transcription of several of these, including *FLOWERING LOCUS T* (*FT*), *SQUAMOSA PROMOTER BINDING PROTEIN-LIKE 15* (*SPL15*), *SUPPRESSOR OF OVEREXPRESSION OF CONSTANS 1* (*SOC1*) and *SEPALLATA 3* (*SEP3*). MADS box transcription factors are proposed to bind DNA as dimers and tetramers [13, 14], and FLC interacts with and binds to a subset of its targets in complexes with the related protein SHORT VEGETATIVE PHASE (SVP) [7, 10]. Mutants at *SVP* are early flowering and exhibit increased levels of *SOC1* and *FT* mRNAs [9, 10, 15, 16]. How FLC represses transcription of its targets is not completely clear, but appears to involve modification of histones. FLC and its homologue FLOWERING LOCUS M (FLM) recruit EMBRYONIC FLOWER 1 (EMF1), a plant-specific Polycomb Repressive Complex 1 (PRC1) component [17, 18], to *FLOWERING LOCUS T* (*FT*) in leaf veins to repress its transcription [19]. Moreover, cooperativity between FLC or FLM and PRC1 contributes to maintenance of PRC2-induced trimethylation of lysine-27 at histone H3 (H3K27me3) at *FT* chromatin, most probably through the activity of the histone methyltransferase CURLY LEAF (CLF) and the H3K27me3-associated protein LIKE HETEROCHROMATIN PROTEIN1 (LHP1) [19]. Additionally, the JmjC domain-containing trimethyl histone H3 lysine-4 demethylase JUMONJI14/PKDM7B (JMJ14/PKDM7B) also associates with PRC1 to further antagonize the active chromatin state at *FT* [20, 21].

Other targets of FLC and SVP that are involved in floral induction encode the MADS-box transcription factor SOC1 and the plant-specific transcription factor SPL15 [22–27]. Both SOC1 and SPL15 are expressed in the shoot apical meristem where they cooperate at the promoters of target genes such as *FRUITFULL* (*FUL*) and *MIR172b* to activate the basal floral promotion pathway under non-inductive environmental conditions [12, 22, 28]. Interestingly, SOC1 coordinates the recruitment of the histone demethylase RELATIVE OF EARLY FLOWERING 6 (REF6) to the promoter of *FUL* and *MIR172b* to orchestrate the removal of the H3K27me3 mark and activate transcription [22]. Furthermore, SPL15 activity is repressed post-transcriptionally by miR156 and post-translationally through its physical interaction with the gibberellin (GA)-labile DELLA protein REPRESSOR OF GA1-3 (RGA) [22]. By contrast, SPL9, a paralogue of SPL15 that is expressed after floral induction at the periphery of the SAM, requires interaction with DELLA proteins to potentiate its *trans*-activation activity and contribute to the induction of expression of the floral meristem identity gene *APETALA1* (*AP1*) in the low gibberellin context present in the cells that give rise to the floral primordium [29].

Here, we show that *TFS1*, which encodes a B3-type transcription factor that is a member of the REPRODUCTIVE MERISTEM (REM) family [30, 31], constitutes a target of FLC and SVP and that its transcription is repressed through cooperation with PRC complexes during vegetative growth. After floral induction, *TFS1* expression is induced at the periphery of the SAM by SOC1 and the age-regulated transcription factor SPL9 through coordinated recruitment of the histone demethylase REF6 and the chromatin remodeler BRAHMA (BRM) [32–34]. This analysis deepens our understanding of the mechanism by which FLC represses the floral network and indicates the importance of antagonistic histone modifications mediated by different MADS box complexes on common target genes.

## Results

### *TFS1* is a target of the floral repressors FLC and SVP

**Fig 1:**
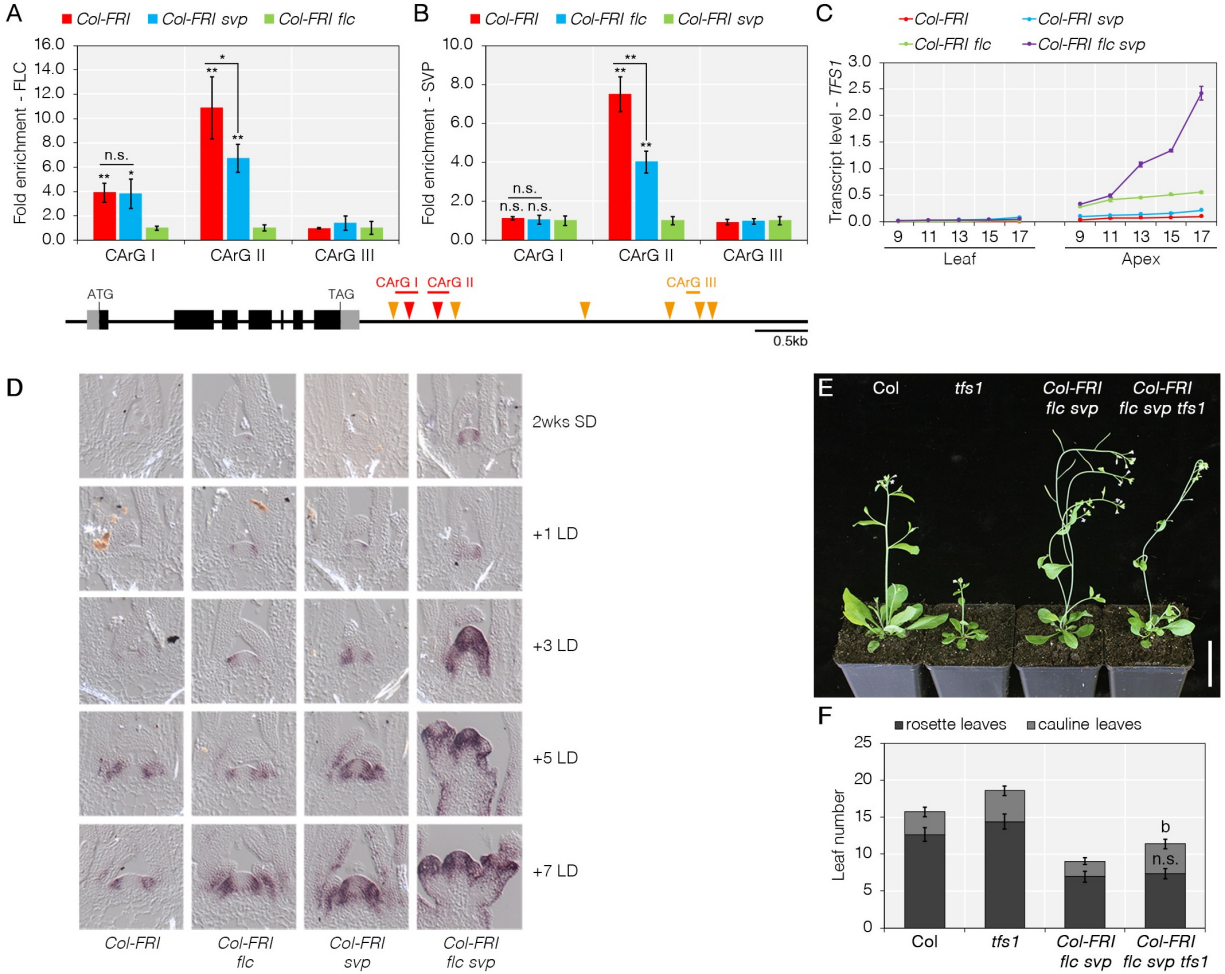
*TFS1* is directly regulated by FLC and SVP during floral induction. A and B) Analysis of FLC (A) and SVP (B) binding to *TFS1* by ChIP-qPCR. Grey and black boxes depict UTR and exons, respectively. Orange and red triangles indicate CArG-boxes with CCW(6)GG and CCW(6)RG consensus, respectively. Plant material used and replicates performed are described in Methods. Values were scaled to set value of CArG-box I in *Col-FRI flc svp* to 1. Statistical significance was calculated using Student’s *t*-test against *Col-FRI flc* (A) and *Col-FRI svp* (B). Also for CArG II the significance of the difference in enrichment between Col-FRI and Col-FRI svp was tested. *P < 0.05, **P < 0.01, n.s. *P* > 0.05. C) Increased expression of *TFS1* in apices of *Col-FRI flc svp* plants by RT-qPCR. Numbers on X-axis indicate number of long-days (LD) for which plants were grown prior to RNA extraction. D) Spatial pattern of expression of *TFS1* assessed by *in situ* hybridization during floral transition. E and F) Representative photo of plants of the illustrated genotypes 28 days after germination under LDs (E) and their respective flowering time (F). Statistical significance was calculated using Student’s *t*-test; *P* < 0.01.

To further define the regulatory network controlling floral transition at the shoot apex, recently published ChIP-Seq and tissue-specific RNA-Seq datasets were examined to identify genes that are expressed specifically at the shoot apex and are bound by the floral repressor transcription factors FLC and SVP [6–8]. Cross-referencing these datasets identified the gene encoding the B3-type transcription factor TARGET OF FLC AND SVP1 (TFS1), which was formerly annotated as REPRODUCTIVE MERISTEM 17 (REM17) [30, 31]. A phylogenetic analysis revealed that *TFS1* is part of a gene family including *REDUCED VERNALIZATION RESPONSE1* (*VRN1*), *VERDANDI* (*VDD*) and *VALKYRIE* (*VAL*), and that *REM18* and *REM19* are the closest homologs of *TFS1* within a sub-branch of the phylogenetic tree (S1A Fig). To verify the binding of FLC and SVP at *TFS1*, chromatin-immunoprecipitation (ChIP) analysis was performed using chromatin extracted from the aerial parts of 15-day old plants grown under inductive long days (LDs) and using antibodies that were raised against FLC [8] and SVP (S1B Fig, S3 and S4 Tables, Methods). In agreement with the ChIP-seq data, specific enrichment of a fragment that encompasses the putative CArG-boxes, designated as CArG-box II, located at the 3’ end of *TFS1* was detected after ChIP of FLC or SVP (Fig 1A and 1B). Moreover, the ChIP-qPCR analyses also demonstrated a mutual co-operation between these floral repressors at *TFS1*, because binding of FLC and SVP was enhanced in the presence of the other protein (Fig 1A and 1B), as reported previously for several other targets of these transcription factors [7, 10].

To further characterize the regulation of *TFS1* by FLC and SVP, the abundance of *TFS1* mRNA in leaves and apices was tested by RT-qPCR in Col-*FRI flc-3*, *Col-FRI svp-41* and *Col-FRI flc-3 svp-41* mutants as well as *Col-FRI* wild-type. *TFS1* mRNA was exclusively detected at the shoot apex and its abundance was increased throughout a developmental time course in the single mutants compared to wild-type and was strongly increased in the double mutant (Fig 1C, S1C Fig). Furthermore, *TFS1* expression was increased at the shoot apex, but not in leaves, after *Col-FRI* plants were exposed to vernalisation and returned to normal growth temperature (S1D Fig), consistent with the repression of *TFS1* transcription by FLC. To determine whether the spatial pattern of expression of *TFS1* differs in *Col-FRI flc-3* and *Col-FRI svp-41* mutants compared to *Col-FRI*, *in-situ* hybridisation analysis of the shoot apex was performed during floral transition after transfer of plants from short days (SDs) to inductive LDs (Fig1D, S1E Fig). The Col-*FRI* and Col-*FRI flc-3* plants grown for 2 wks under SD were still vegetative (Fig 1D), but *TFS1* expression level was clearly elevated in *Col-FRI flc-3* (S1C Fig), indicating that FLC represses *TFS1* expression even in the vegetative stage. After transfer to LDs, *TFS1* mRNA appeared at the periphery of the SAM in the *Col-FRI* wild-type. This spatial pattern was not changed in the *Col-FRI svp-41*, *Col-FRI flc-3* or *Col-FRI flc-3 svp-41* mutants, but the mRNA appeared more rapidly after transfer to LDs in the mutants than in *Col-FRI* wild-type (Fig 1D, S1C Fig). Therefore, *TFS1* transcription is induced during floral transition, while the timing and amplitude of its expression are modulated by SVP and FLC.

The temporal expression pattern of *TFS1* coincided with the transition to flowering, so the flowering time of *tfs1* mutants was determined under inductive and non-inductive conditions. Interestingly, *tfs1-1* mutants flowered significantly later than wild-type plants under both conditions, suggesting that *TFS1* is involved in promoting floral transition (Fig 1E and 1F, S2A–S2F Fig). The role of *TFS1* was confirmed by transgenic complementation of *tfs1-1* (see later), and by showing that a second allele (*tfs1-2*) caused a similar late-flowering phenotype under LDs (S2E and S2F Fig). The *tfs1-1* mutation also delayed flowering in the *Col-FRI flc-3 svp-41* background, supporting the idea that TFS1 acts downstream of FLC and SVP to promote flowering (Fig 1E and 1F). In addition, the *Col-FRI flc-3 svp-41 tfs1-1* plants showed impaired flower development, suggesting redundancy between these genes in flower and inflorescence development (S2G and S2H Fig). Overall, these results demonstrate that *TFS1* is a direct target of FLC and SVP and is specifically expressed at the periphery of the SAM during floral transition to promote flowering and floral development.

### Histone modification of *TFS1* through the activity of POLYCOMB REPRESSIVE COMPLEX 2 is dependent on FLC/SVP

**Fig 2:**
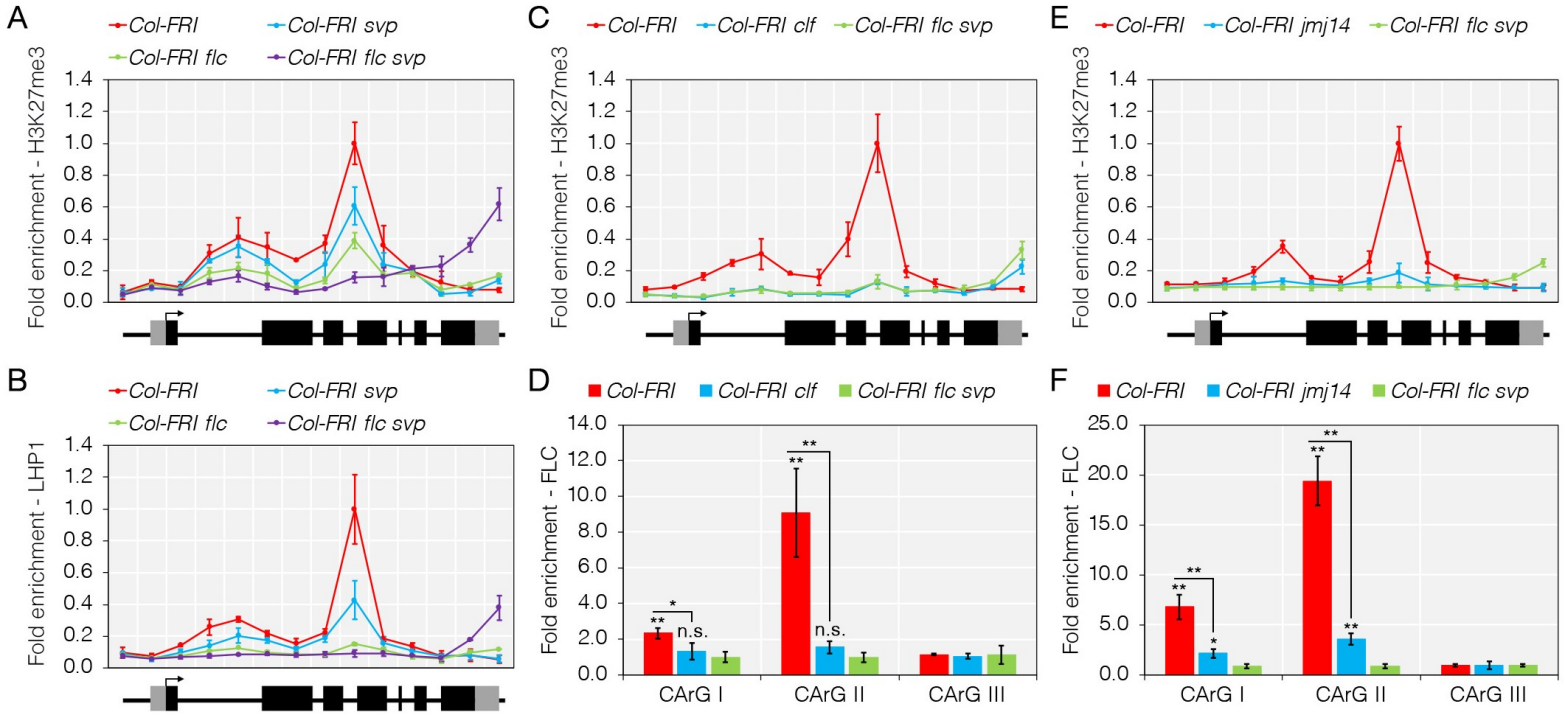
Binding of FLC and SVP to *TFS1* is associated with accumulation of H3K27me3. A) Accumulation of H3K27me3 at *TFS1* is reduced in *Col-FRI flc svp* plants. B) LHP1 is associated with a region of *TFS1* that heavily accumulates H3K27me3. C) Accumulation of H3K27me3 at *TFS1* is reduced in *Col-FRI clf* mutants. D) Binding of FLC to *TFS1* is reduced in *Col-FRI clf* plants. E) Accumulation of H3K27me3 at *TFS1* is reduced in *Col-FRI jmj14* mutants. F) Binding of FLC to *TFS1* is reduced in *Col-FRI jmj14* plants. A to C and E, values were scaled to set value of first primers in *Col-FRI* to 1. D and F, values were scaled to set value of CArG-box I in *Col-FRI flc svp* to 1. Statistical significance was calculated against *Col-FRI flc svp* using Student’s *t*-test as described in Fig 1A and 1B; *P < 0.05, **P < 0.01, n.s. *P* > 0.05.

Transcriptional repression by FLC and SVP has been linked to activity of Polycomb Repressive Complex (PRC) 2 (PRC2) [10, 19]. PRC2 catalyzes the methylation of histone 3 (H3) at lysine 27 (H3K27me3) and is associated with the repression of transcription [35]. Therefore, whether *TFS1* is subjected to PRC-mediated regulation in a FLC and SVP dependent manner was tested by performing ChIP-qPCR using chromatin extracted from 15-day old plants grown under LDs and antibodies directed against H3K27me3 or H3K4me3 (S3 Table). H3K27me3 was detected in the gene body of *TFS1* in *Col-FRI* plants at much higher levels than in *Col-FRI flc-3*, *Col-FRI svp-41* and *Col-FRI flc-3 svp-41* mutants (Fig 2A). However, in *Col-FRI flc-3 svp-41* plants an additional peak in H3K27me3 levels was detected close to the transcriptional termination site of *TFS1* (Fig 2A, S3A Fig). Furthermore, a commercially available antibody (S3 Table) was used for ChIP-qPCR of LHP1, which is frequently found associated with H3K27me3 marked chromatin [36, 37], and the protein was detected at *TFS1* in a similar pattern to H3K27me3 (Fig 2B). Therefore, H3K27me3 and LHP1 are present in the gene body of *TFS1* in a FLC and SVP dependent manner correlating with reduced transcription of *TFS1*.

In contrast to H3K27me3, K4-trimethylated H3 (H3K4me3) is present at genes that are actively transcribed [38]. Enrichment of H3K4me3 was detected close to the transcriptional start site (TSS) of *TFS1* in the *Col-FRI flc-3*, *Col-FRI svp-41* and *Col-FRI flc-3 svp-41* mutants, whereas it was not present at the gene in *Col-FRI* plants (S3B Fig). These data are in agreement with previous reports of the dynamic and antagonistic relationship between H3K27me3 and H3K4me3 during development and the floral transition [35, 39]. Therefore, under these conditions the dynamic change in these chromatin marks at *TFS1* correlates with the repression of *FLC* transcription, the induction of *TFS1* and the transition to flowering.

The PRC2 mutation *curly leaf* (*clf*), which impairs the activity of an enzyme that catalyses H3K27me3 deposition [40], partially suppresses the late-flowering phenotype of *Col-FRI* plants [41]. Strikingly, early flowering *Col-FRI clf-2* mutant plants expressed highly elevated levels of *TFS1* mRNA in the apex, while still expressing high levels of *FLC* and *SVP* mRNA (S3D–S3F Fig) [41, 42]. These observations supported the idea that PRC2 might contribute to transcriptional repression of FLC target genes such as *TFS1*. To examine this possibility, ChIP analyses were performed to test for H3K27me3 enrichment across the coding region of *TFS1* in *Col-FRI clf-2* and *Col-FRI flc-3 svp-41* mutant plants. In agreement with the functional role of CLF in the deposition of H3K27me3, a strong reduction in this mark at *TFS1* was detected in *Col-FRI clf-2* and this was similar to the reduction observed in *Col-FRI flc-3 svp-41* mutant plants when compared to wild-type *Col-FRI* (Fig 2C). In contrast, ChIP analyses for H3K4me3 at *TFS1* showed higher enrichment patterns in *Col-FRI clf-2* as well as in *Col-FRI flc-3*, *Col-FRI svp-41* and *Col-FRI flc-3 svp-41* mutants than in wild-type *Col-FRI* (S3B and S3C Fig). These data indicate that reduction in H3K27me3 at *TFS1* in *Col-FRI clf-2* mutants correlates with an increase in H3K4me3, consistent with the antagonistic role of these marks during floral transition. Finally, to determine whether the reduction in H3K27me3 levels at *TFS1* in *Col-FRI clf-2* plants correlated with reduced FLC binding, ChIP analyses were performed for FLC. FLC binding was strongly compromised in *Col-FRI clf-2*, indicating that FLC binding requires and is sustained by PRC2 function (Fig 2D).

SVP and FLC interact respectively with LHP1 and the Polycomb Repressive Complex 1 protein EMBRYONIC FLOWER (EMF1) [18, 19, 43, 44], suggesting a link between PRC function and the activity of these floral repressors. Furthermore, a complex including LHP1, EMF1 and the H3K4me3 demethylase JMJ14/PKDM7B, has been described to play roles related to PRC1, including delaying flowering in non-inductive photoperiods [19–21]. These observations, together with the result described above that H3K4me3 levels are lower at *TFS1* in the presence of SVP and FLC (S3B Fig), suggested that the H3K4me3 demethylase activity of JMJ14 might be required for FLC and PcG mediated repression of *TFS1*. To test this idea, ChIP-qPCR analyses of H3K27me3 were performed on *TFS1* in *Col-FRI jmj14-2* mutants. In these plants the enrichment levels of the repressive mark H3K27me3 were strongly reduced compared to *Col-FRI* wild-type (Fig 2E). By contrast, the active chromatin mark H3K4me3 was increased at *TFS1* in *Col-FRI jmj14-2* compared to *Col-FRI* and was present at a similar level as in *Col-FRI flc-3 svp-41* mutants (S3C Fig). Consistent with the increased levels of H3K4me3, *Col-FRI jmj14-2* also showed higher mRNA levels of *TFS1* in apices, but did not affect the expression of *SVP* or *FLC* (S3G–S3I Fig). In support of the notion that FLC binding to *TFS1* requires PRC2 activity and higher levels of H3K27me3 (Fig 2D), reduced binding of FLC to CArG-box II at *TFS1* was also detected in *Col-FRI jmj14-2* plants, although *FLC* mRNA level was unaffected (Fig 2F, S3H Fig). Collectively, these data suggest that for FLC mediated transcriptional repression of *TFS1*, the recruitment of PRC2 and deposition of H3K27me3 are required, and that these can also be inhibited by increasing the levels of H3K4me3 through mutation of the JMJ14 demethylase.

### FLC and SVP mediated repression of *TFS1* is coupled to a gene loop

**Fig 3:**
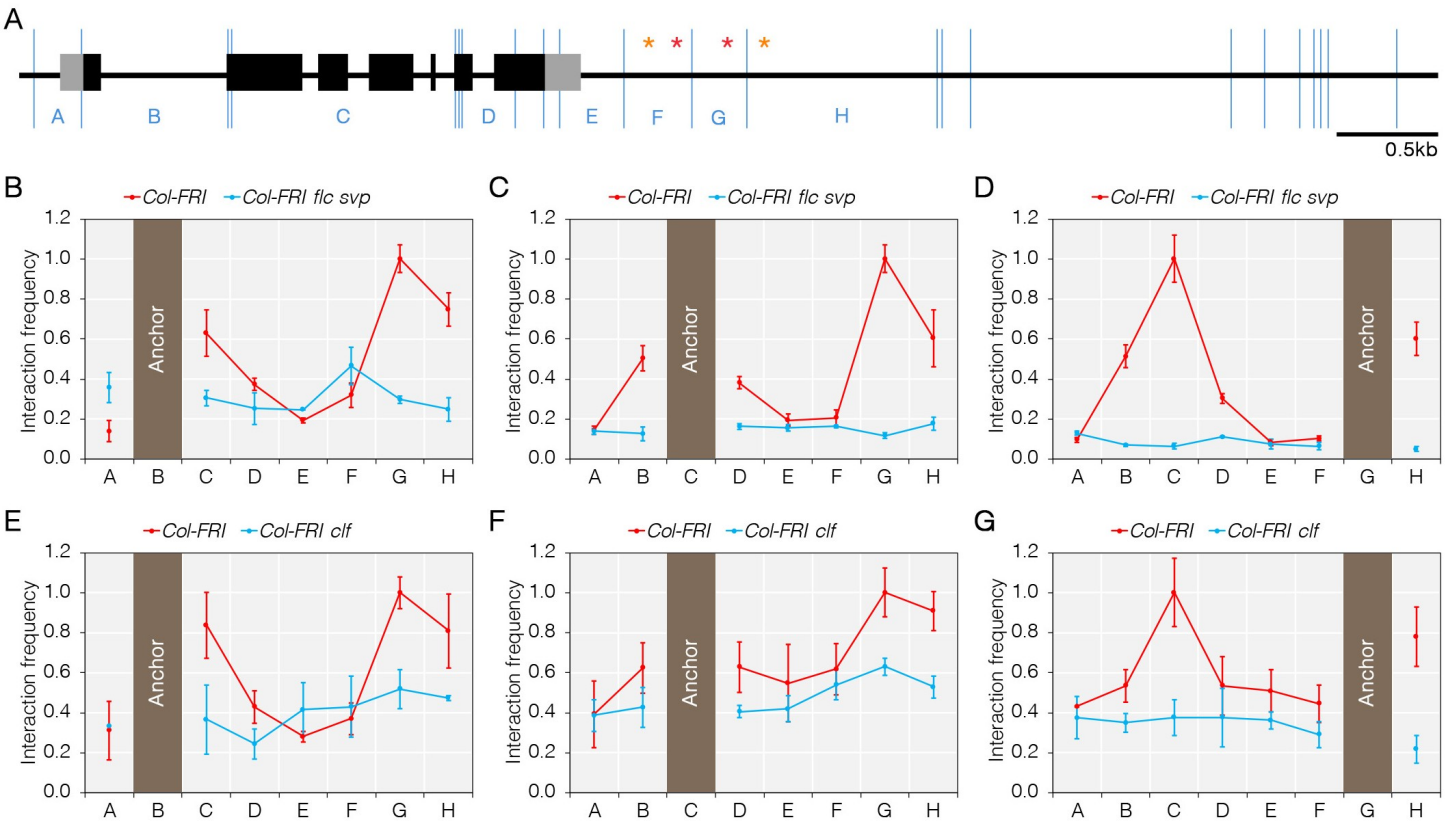
FLC and SVP are associated with looping between the 3’ end of *TFS1* and gene body. A) High resolution digestion map of the *TFS1* locus by the enzyme Sau3AI. Primers were designed on 8 restriction fragments as indicated (A to H). Restriction sites are highlighted with blue vertical lines. CArG-boxes are presented as red and orange asterisks. Exons are indicated as black boxes and 5’ and 3’ UTR are indicated as grey boxes. B to D) DNA looping between coding region and 3’ end of *TFS1* is reduced in *Col-FRI flc svp* plants, but not in *Col-FRI*. E to G) DNA looping between coding region and 3’ end of *TFS1* is reduced in *Col-FRI clf* plants, but not in *Col-FRI*. Plants were collected for 3C experiments after 14 LD at ZT4. Values were scaled to set the highest interaction value per fixed primer to 1. The position of the fixed primers is indicated by a filled column designated as ‘Anchor’.

The observations that FLC and SVP bind 3’ of the *TFS1* stop codon (Figs 1A, 1B, 2D and 2F) and that they interact with a PRC complex and LHP1 [19, 43] that are associated with the gene body of *TFS1* (Fig 2B), suggested that a chromosomal loop might form between the 3’ distal region and the gene body. To test for the presence of such a loop, a chromosome conformation capture (3C) assay was performed. Indeed, in Col-FRI plants fragments B, C and D (middle region) were found to interact with the 3’ end (G and H) of *TFS1*, suggesting that a ‘locked’ DNA loop was formed (Fig 3). The presence of this loop was then tested in different mutants to determine whether it required FLC/SVP and PcG function. In *Col-FRI flc-3 svp-41* mutants, the interaction between the middle region and the 3’ end was strongly reduced compared to *Col-FRI*, indicating that FLC and SVP are required for the formation of the ‘locked’ DNA loop (Fig 3A–3D). To define the contribution of PRC2 in the formation of this loop at *TFS1*, *Col-FRI clf-2* plants were examined. In this genotype, the loop appeared significantly weaker than in *Col-FRI* plants, indicating that CLF activity is required to support the formation of the ‘locked’ DNA loop at *TFS1* (Fig 3E–3G). These data indicate therefore that transcriptional repression of *TFS1* by FLC and SVP is associated with the formation of a chromatin loop that requires FLC and SVP binding at the 3’ end of the gene and high levels of H3K27me3 within the gene body.

### Activation of *TFS1* transcription by SOC1

**Fig 4:**
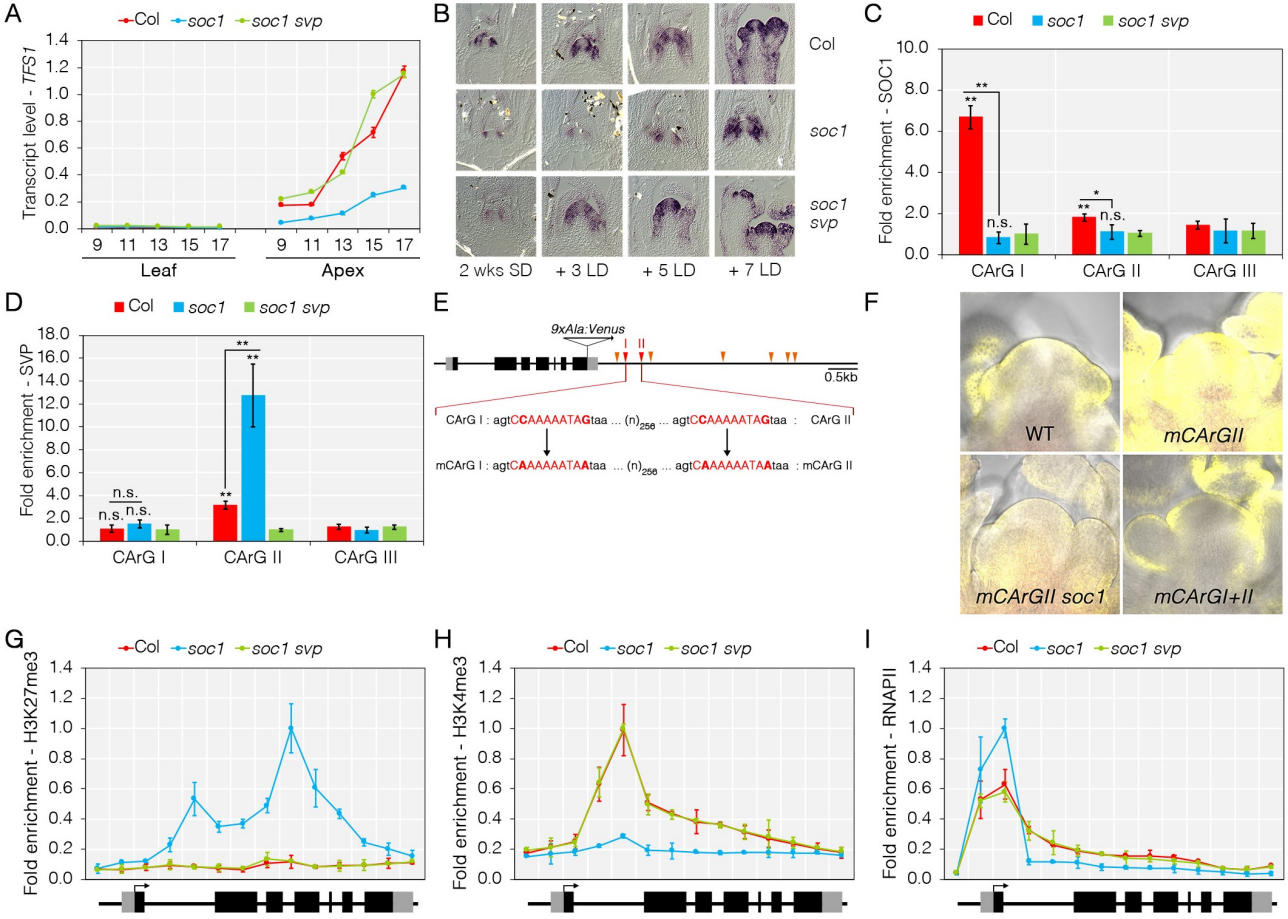
*TFS1* is activated during floral transition by direct binding of SOC1. A) *TFS1* transcript is reduced in apices of *soc1* mutants during floral transition. Numbers on X-axis indicate number of long-days (LD) for which plants were grown prior to RNA harvest. B) Spatial pattern of expression of *TFS1* in *soc1* and *soc1 svp* mutants. C) SOC1 binds to the 3’ end of *TFS1* as detected by ChIP-qPCR. Statistical significance was calculated against *soc1 svp*. D) SVP binding to the 3’ end of *TFS1* is reduced in the presence of SOC1. Statistical significance was calculated against *soc1 svp*. E) Model of *TFS1* genomic region and the mutations introduced into CArG-boxes. CArG-box with consensus sequence CW6RG (red triangle) and CW6GG (orange triangle) are shown. F) SOC1 activates *TFS1* through CArG box I at the 3’ end of *TFS1*. Images were taken from 17 LD-grown seedlings. G) H3K27me3 levels at *TFS1* are increased in *soc1* mutants compared to Col. H) H3K4me3 levels are reduced at *TFS1* in *soc1* mutants compared to Col. I) RNA PolII stalls at the 5’ end of *TFS1* in *soc1* mutants. G to I, values were scaled to set value of the first primers in Col to 1. Statistical significance was calculated using Student’s *t*-test; *P < 0.05, **P < 0.01, n.s. *P* > 0.05.

In a genome-wide study of binding sites of the MADS-box protein SOC1, a site at the 3’ end of *TFS1* was detected [28]. To determine whether *TFS1* is regulated by SOC1, *TFS1* transcript abundance was tested by RT-qPCR using RNA extracted from leaves and apices in *soc1-2*, *soc1-2 svp-41* and Col genotypes. *TFS1* mRNA levels were much lower in apices of *soc1-2* mutants than Col, but were largely restored to Col levels in *soc1-2 svp-41* double mutants (Fig 4A). To determine the spatial pattern of *TFS1* expression in *soc1-2* and *soc1-2 svp-41*, *in-situ* hybridisations were performed on apices during floral transition. The overall spatial expression pattern was similar in Col and *soc1-2 svp-41*, however, a significant delay in *TFS1* expression at the periphery of the SAM was observed in *soc1-2* (Fig 4B).

ChIP analyses were then performed with antibodies that were directed against endogenous SOC1 and SVP (S1B Fig, S3 Table, [22]). To verify the results of the previous report on the genome-wide study for SOC1, ChIP-qPCR was performed and detected SOC1 binding in the region of CArG-box I (CArGI), which is located at the 3’ end of *TFS1* (Figs 1A and 4C). The ChIP-qPCR experiment with SVP generated a specific enrichment in the region of CArG-box II (CArGII) in Col, and this was enhanced in the *soc1-2* mutant, suggesting that SOC1 reduces SVP recruitment to *TFS1* (Fig 4D). Similarly, Dexamethasone (DEX)-induced translocation of SOC1:GR into the nucleus in *35S*::*SOC1*:*GR* plants caused higher *TFS1* transcription and increased binding of SOC1 to CArGI as well as reduced binding of SVP to CArGII (S4A–S4C Fig). Therefore, binding of SOC1 to the 3’ end of *TFS1* occurs during floral transition, whereas SVP binds during vegetative development, and is in agreement with their observed roles in the transcriptional regulation of *TFS1*.

To test *in vivo* whether the CArG-boxes identified within the ChIP-qPCR amplicons are responsible for the regulation of *TFS1* by SOC1 and SVP, a *TFS1*::*TFS1*:*9xAla-Venus* (*TFS1*::*TFS1*:*9AV*) gene fusion was constructed that contained the entire intergenic region flanking *TFS1* on the 5’ and 3’ sides (Fig 4E). This gene fusion complemented the *tfs1-1* mutant phenotype (S5A and S5B Fig) and in the transgenic plants VENUS signal was detected at the periphery of the SAM in a similar pattern as observed by *in situ* hybridization of *TFS1* mRNA (Figs 1D and 4F, S4E Fig). Also, the confocal imaging of the TFS1:9xAla-Venus fusion protein indicated that it predominately localized in the cytosol of slowly dividing meristematic cells (S6A and S6B Fig) while treatment with leptomycin B (LMB), which impairs the activity of nuclear exportin [45], suggested that this was due to active export from the nucleus (S6C Fig). However, in actively dividing cells in young sepals the VENUS signal appeared to be nuclear (S6D to S6G Fig), suggesting the nuclear accumulation and presumably the activity of TFS1 may be closely related to cell division.

The CArG-boxes identified in the ChIP amplicons were then mutated in this gene fusion construct. Two mutant plasmids were generated in which CArGII or both CArGI and CArGII were mutated (Fig 4E). Several independent transformants carrying each construct were analysed (Fig 4F, S4E–S4H Fig). Transformants harbouring the mCArGII construct displayed a stronger and broader VENUS signal than those carrying the wild-type construct, whereas the mCArGI+II construct conferred a VENUS signal that was similar to the wild-type construct (Fig 4F, S4E–S4G Fig). The relative strength of these mutant constructs was supported by RT-qPCR analysis performed on RNA extracted from apices of the transgenic plants (S4H Fig). Furthermore, the strong signal of the mCArGII construct was greatly reduced when it was introduced into the *soc1-2* mutant by crossing (Fig 4F). The low level of TFS1:9AV expression detected in the *soc1 TFS1*::*TFS1*:*9AV* mCArGII and *TFS1*::*TFS1*:*9AV* mCArGI+II plants was consistent with their delayed flowering time compared to *TFS1*::*TFS1*:*9AV* plants (S5A–S5H Fig). Taken together, these observations were consistent with the transcriptional profile of *TFS1* in Col and *soc1-2 svp-41* (Fig 4A), and supported the proposal that SOC1 activates and SVP represses transcription of *TFS1* at least partly through binding to CArG-box I and CArG-box II, respectively.

Consistent with the role of SOC1 in promoting *TFS1* transcription, increased levels of the repressive mark H3K27me3 were detected in aerial parts of 15-day old *soc1-2* mutants across the *TFS1* gene body (Fig 4G). Furthermore, in DEX-induced *35S*::*SOC1*:*GR* plants, H3K27me3 levels were reduced following binding of SOC1 (S4D Fig). Additionally, the presence of the active chromatin mark H3K4me3 and of RNA polymerase II (RNAPII) were monitored along the transcribed region of *TFS1* in *soc1-2* mutants. ChIP-qPCR analysis revealed that H3K4me3 levels were strongly reduced in *soc1-2* mutants, similar to those observed in *Col-FRI* wild-type (Figs 2A and 4H). Also, the ChIP profile of RNAPII demonstrated enrichment throughout the transcribed region of *TFS1* in Col and *soc1-2 svp-41*. By contrast, in *soc1-2* mutants the loss of RNAPII enrichment was most apparent in regions of the gene body (Fig 4I), which is reminiscent of inactive genes that display a poised RNAPII machinery at promoter regions.

Overall, these experiments demonstrate that SOC1-induced activation of *TFS1* transcription is invoked by eviction of SVP and reduction in H3K27me3 as well as by releasing RNAPII to transcribe the gene.

### SOC1 recruits REF6 and BRM to *TFS1* to activate transcription

**Fig 5:**
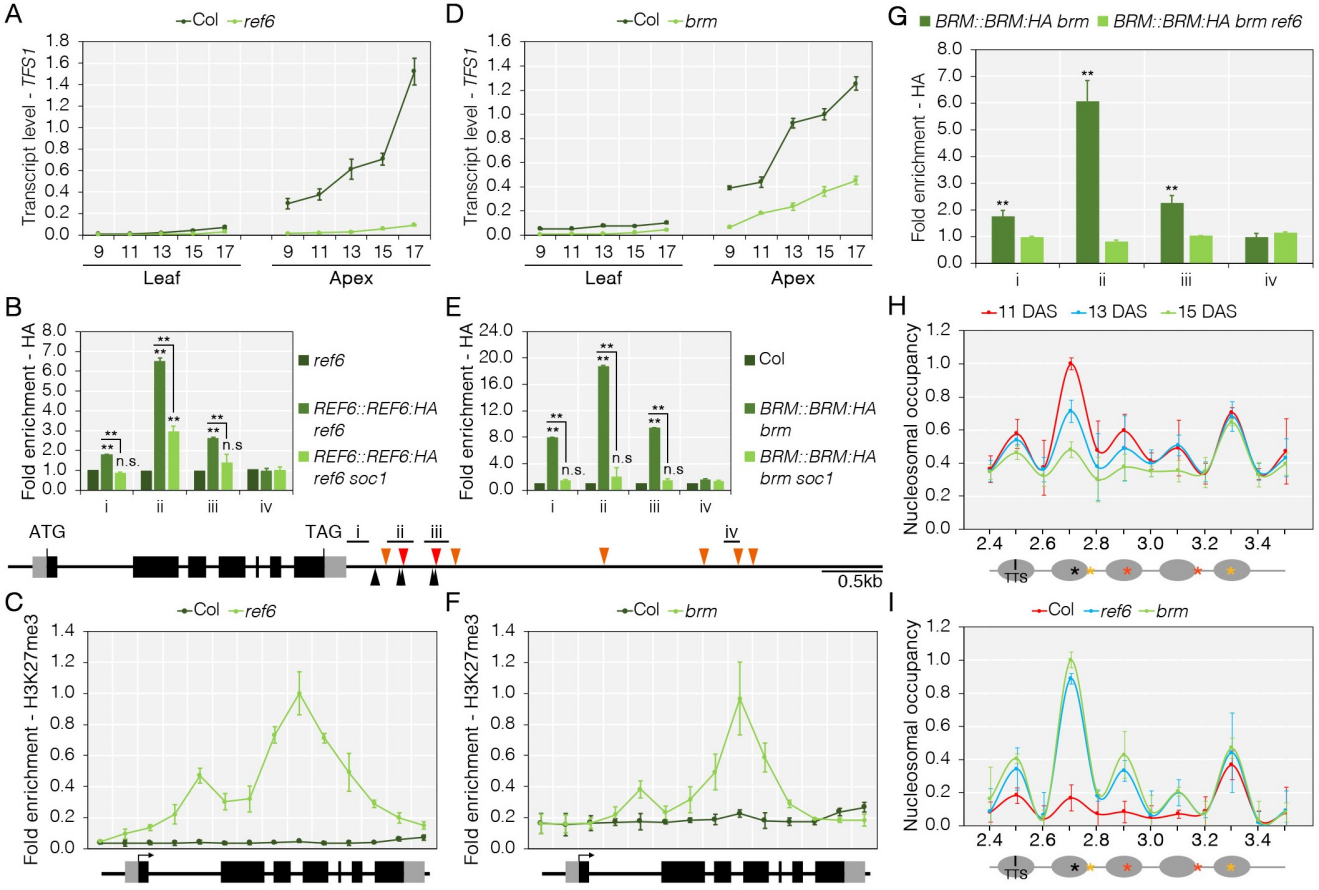
Activation of *TFS1* transcription requires direct recruitment of REF6 demethylase and BRAHMA chromatin remodelling ATPase. A) Reduced *TFS1* mRNA levels in *ref6* mutants during floral transition compared to Col. B) Top: REF6 recruitment to *TFS1* depends on *SOC1*. Bottom: *TFS1* gene model highlighting putative REF6 binding sites. CArG-box are indicated in red and orange triangles and black triangles show putative binding sites for REF6. C) REF6 is required to remove H3K27me3 at *TFS1*. D) Reduced *TFS1* mRNA levels in *brm* during floral transition compared to Col. E) BRM recruitment to *TFS1* depends on *SOC1*. F) BRM is required to remove H3K27me3 at *TFS1*. Values were scaled to set the value of the first primer set in Col to 1. G) BRM binding to 3’ end of *TFS1* requires REF6. Values were scaled to set peak value of ii in *BRM*::*BRM*:*HA brm* to 1. Statistical significance was calculated against *BRM*::*BRM*:*HA brm ref6*. H) Nucleosome positioning at 3’end of *TFS1*. MNase digestion followed by tiled oligo qPCR (MNase-qPCR) to monitor nucleosome positioning at 3’ end of *TFS1* in 11-, 13- and 15-day-old LD-grown Col plants. Values were scaled to set highest peak value to 1. CArG-boxes are indicated in red and orange asterisks and black asterisk shows putative SPL-binding sites. TTS, transcriptional start site. I) Nucleosome positioning at 3’end of *TFS1* in Col, *ref6* and *brm* plants that were grown for 15LD. Values were scaled to set highest peak value to 1. In A and D, Numbers on x-axis indicate number of long-days (LD) for which plants grown prior to harvest. In B and E, Values were scaled to set value of amplicon i to 1 in *ref6* and Col, respectively. Statistical significance was calculated against *ref6* (B) and Col (E) using Student’s *t*-test; **P < 0.01, n.s. *P* > 0.05.

The histone demethylase REF6 and the chromatin remodeler BRM physically interact to antagonize PcG proteins at target loci, and both proteins were detected at the 3’ end of *TFS1* in a genome-wide study [33, 34, 46]. Furthermore, REF6 and SOC1 co-purified in the same complex, which was required to facilitate transcriptional activation of target genes through the removal of the repressive histone mark H3K27me3 [22, 47]. Thus, to understand the effects of REF6 and BRM on *TFS1* transcription, *TFS1* transcript abundance was monitored by RT-qPCR in *ref6-1* and *brm-1* mutants grown for 9 to 17 days in LDs. Throughout the time-course, the transcript profile of *TFS1* was not changed in leaves of *ref6-1* or *brm-1* mutants compared to Col, but a dramatic reduction in *TFS1* transcript abundance was detected in apices of both mutants (Fig 5A and 5D). Therefore, REF6 and BRM are required for the activation of *TFS1* in apices.

To validate the previously reported binding of REF6 and BRM to *TFS1* [46], ChIP-qPCR analyses were performed using *REF6*::*REF6*:*HA ref6-1* and *BRM*::*BRM*:*HA brm-1* transgenic lines [47, 48]. Binding of REF6:HA and BRM:HA to sites located at the 3’ end of *TFS1* was detected and these sites flank CArGI, to which SOC1 binds (Fig 5B and 5E). To understand whether association of REF6 and BRM with chromatin is dependent on SOC1 (Fig 4C), the *soc1-2* mutation was introduced into the *REF6*::*REF6*:*HA ref6-1* and *BRM*:*BRM*:*HA brm-1* transgenic lines by genetic crossing. A strong reduction in binding of REF6:HA and BRM:HA was detected by ChIP-qPCR in *soc1-2* mutants, indicating that SOC1 supports REF6 and BRM binding to the 3’-end of *TFS1* (Fig 5B and 5E). Additionally, the *BRM*:*BRM*:*HA brm-1* transgene was introduced into the *ref6-1* mutant to study binding behaviour of BRM:HA to the 3’ end of *TFS1*. Chromatin association of BRM:HA was strongly reduced in *ref6-1* compared to Col (Fig 5G), which further corroborated the idea that REF6 is required for BRM recruitment.

REF6 is a H3K27me3 demethylase [47] and BRM and REF6 appeared to act as direct activators of *TFS1* transcription, so H3K27me3 levels were tested at *TFS1* in the respective mutants. Compared to Col, increased H3K27me3 levels were detected by ChIP-qPCR along the *TFS1* genomic locus in *ref6-1* and *brm-1* mutants. The pattern of increase of H3K27me3 was identical in both mutants and consistent with the observed increase in *soc1-2* mutants (Figs 4G, 5C and 5F). In summary, these findings suggest that the histone demethylase REF6 and the chromatin remodeler BRM are required to activate transcription of *TFS1* in association with SOC1.

To test whether BRM recruitment leads to activation of *TFS1* through changes in chromatin accessibility, limited Micrococcal nuclease (MNase) digestion followed by tiled oligo qPCR was employed to identify well-positioned nucleosomes near the SOC1, REF6 and BRM bound site at the 3’ end of *TFS1*. The MNase-qPCR analysis identified in *ref6-1* and *brm-1* mutants a nucleosome at a position that encompasses the binding site for SOC1 and this nucleosome was destabilized in Col (Fig 5H and 5I). Therefore, SOC1 appears to increase chromatin accessibility and transcription of *TFS1* through recruitment of REF6 and BRM.

### Co-operativity between SOC1 and SPL9 transcription factors in the activation of *TFS1*

**Fig 6:**
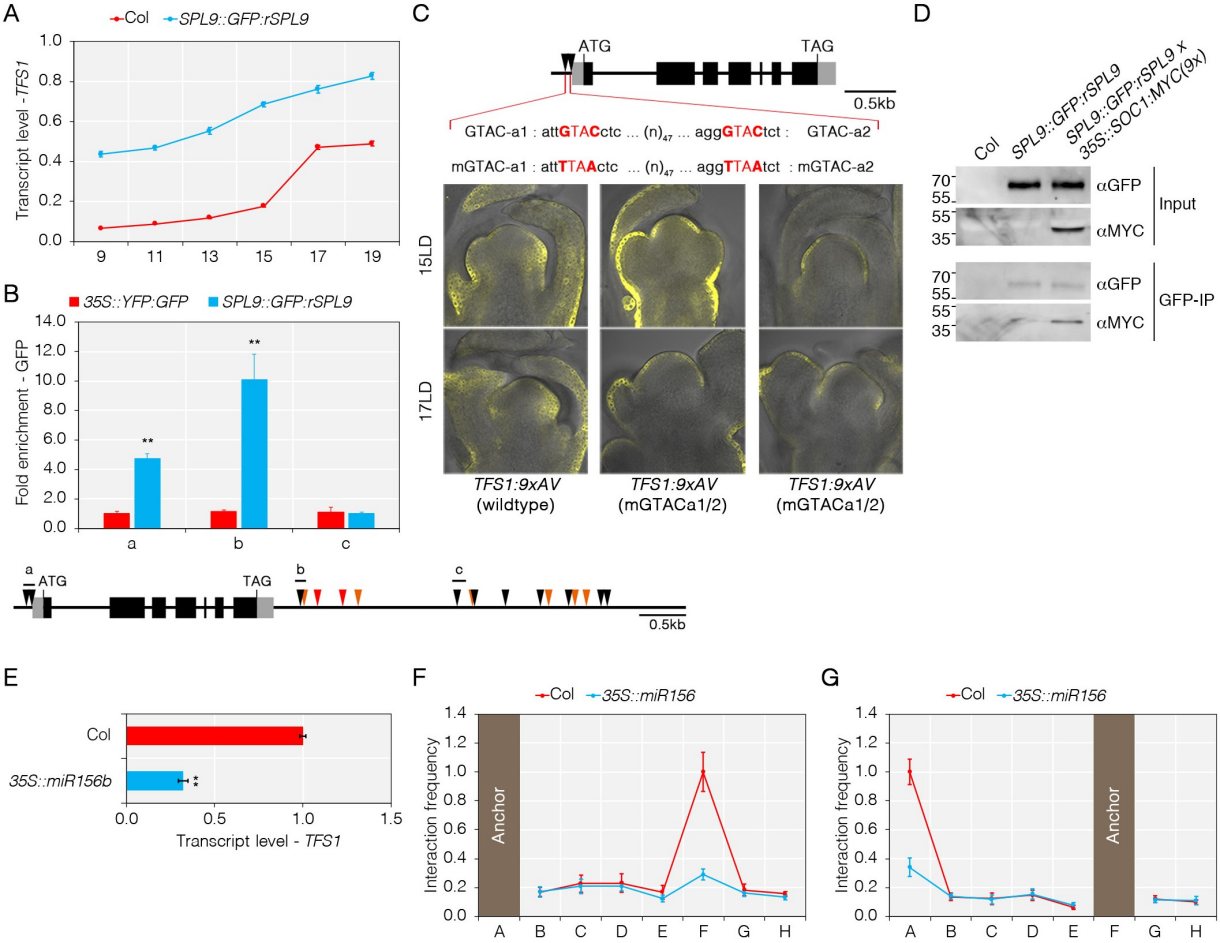
Cooperativity between SPL9 and SOC1 in the activation of *TFS1*. A) Increased expression of *TFS1* is enhanced by rSPL9. Numbers on X-axis indicate number of long-days (LD) for which plants were grown prior to harvest. B) Top: SPL9 binds to the 5’ and 3’ end of *TFS1*. Bottom: Cartoon of potential SPL9 binding sites and amplicons used for ChIP-PCR. Binding sites for SPL9 are depicted in black and CArG-boxes in red and orange triangles. Values were scaled to set value of amplicon a in 35S::YFP:GFP to 1. Statistical significance was calculated using Student’s *t*-test; **P < 0.01. C) Top: Model of *TFS1* genomic region and the mutations introduced into SPL-binding sites. Bottom: Mutation of two putative SPL binding sites reduces *TFS1* expression during floral transition. D) SOC1 co-immunoprecipitates with SPL9. Values on the left of the Western-blots indicated molecular weight (kDa). E) Overexpression of *miR156b* reduces *TFS1* mRNA levels. Statistical significance was calculated using Student’s *t*-test; **P < 0.01. F and G) Overexpression of *miR156b* reduces loop formation between 5’ and 3’ end of *TFS1*.

The spatial and temporal expression patterns of *TFS1* appeared similar to those of *SPL9* [22, 49, 50], which encodes a transcription factor that binds to regulatory sequences in the promoter of the floral meristem-identity gene *APETALA1* (*AP1*) to regulate floral fate [29, 50]. Moreover, SPL15 and SOC1 co-operate to regulate floral commitment under non-inductive conditions [22]. Taken together, the data suggested that SPL9 and SOC1 might cooperate to activate *TFS1*. To test for the molecular effect of SPL9, RNA was extracted from apices of *SPL9*::*GFP*:*rSPL9* to monitor *TFS1* transcript abundance by RT-qPCR. *TFS1* transcript levels were strongly increased in *SPL9*::*GFP*:*rSPL9* plants compared to wild-type (Fig 6A). Next, ChIP-qPCR analysis was employed to test binding of GFP:rSPL9 at the 5’ and 3’ ends of *TFS1* (Fig 6B, S7A and S7B Fig). Consistent with direct activation of *TFS1* by SPL9, fragments at the 5’ and 3’ ends of the gene were enriched after immunoprecipitation of GFP:rSPL9 (Fig 6B, S7A and S7B Fig).

The presence of markers for transcriptional activity at *TFS1*, particularly the Mediator head-module component Med18, RNAPII and H3K4me3, was scored by ChIP-qPCR in different genotypes. Higher enrichment levels of these markers were detected at *TFS1* in *SPL9*::*GFP*:*rSPL9* than in wild-type, supporting that SPL9 activates *TFS1* (S7C–S7F Fig). In contrast, reduced *TFS1* transcript abundance was detected in *spl9-1*, *spl15-1 and spl9-1 spl15-1* mutants (S8A–S8E Fig) and this was accompanied with a reduction in H3K4me3 and an increase in H3K27me3 at the *TFS1* locus (S8F–8H Fig).

Whether the identified binding sites for SPL9 are responsible for *in vivo* regulation of *TFS1* was then examined. To this end, a reporter gene cassette was constructed, mGTACa1/2, in which the two GTAC motifs overlapping with the ChIP-qPCR peak of GFP:rSPL9 at the 5’ end of *TFS1* were mutated (Fig 6C). Transformants harbouring the mGTACa1/2 mutated form were generated and compared by confocal microscopy with plants harbouring a wild-type construct. VENUS fluorescent signal detected at the periphery of the SAM in wild-type was missing in the mGTACa1/2 plants, supporting the idea that SPL9 binds to these sites to activate transcription (Fig 6C, S7G Fig). Surprisingly, however, VENUS fluorescent signal was retained in the epidermis of mGTACa1/2 plants, indicating that expression in these cells likely takes place independently of SPL9 and other SPL transcription factors (Fig 6C, S7G Fig). Additionally, *in-situ* hybridisation indicated that *TFS1* mRNA appeared more rapidly on the flanks of the meristem after transferring *SPL9*::*GFP*:*rSPL9* plants from SDs to LDs than after transferring Col wild-type (S7F Fig). These studies are consistent with SPL9 binding to the 5’ end of *TFS1* to activate transcription at the periphery of the SAM.

In support of the notion of functional cooperativity between SPL9 and SOC1, co-immunoprecipitation of GFP:rSPL9 and SOC1:MYC(9x) was detected in protein extracts from shoot apical tissue of *SPL9*::*GFP*:*rSPL9 35S*::*SOC1*:*MYC(9x)* transgenic lines (Fig 6D). The cooperativity between SPL9 binding at the 5’ end of *TFS1* and SOC1 binding at the 3’ end suggested that DNA loop formation might occur between their binding regions. Therefore, chromosome conformation capture (3C) was employed to test for DNA loop formation in Col and *35S*::*miR156b*, in which several redundant SPL transcription factors are reduced in expression [51] leading to reduced transcription of *TFS1* (Fig 6E). The 3C analyses suggested that interaction between SPL-binding sites located at the 5’ end and the CArG-box predicted to bind SOC1 that is located at the 3’ end of *TFS1* occurred in a SPL9 dependent manner (Figs 4C, 6F and 6G). Together with results described above, the data suggest that SPL9 cooperates with SOC1 to form an ‘active’ DNA-loop that is required for active *TFS1* transcription.

### BRAHMA recruitment by SOC1 is required for SPL9 binding

The chromatin remodeler BRM is recruited to *TFS1* in a SOC1 dependent-manner to increase chromatin accessibility and *TFS1* transcription (Fig 5E and 5I). In addition, SOC1 is required for increased H3K4me3 at *TFS1* (Fig 4H). This chromatin mark is also supported by the COMPASS-like (Complex Proteins Associated with Set1) histone H3 lysine-4 methyltransferase complex component WD40 REPEAT HOMOLOG 5 (WDR5), which associates with the active elongating RNAPII [52]. Therefore, ChIP analyses were performed using commercial antibody (S3 Table) that recognises WDR5a and WDR5b [53] to test WDR5 enrichment at *TFS1* in different genotypes. Consistently, the presence of WDR5 at *TFS1* was decreased across the gene body in *soc1-2*, *brm-1* as well as *spl9-1*, *spl15-1 and spl9-1 spl15-1* mutants (S9A–S9C Fig). Additionally, using commercial antibody (S3 Table) ChIP analyses for the histone variant H2A.Z, which marks both transcriptionally active and inactive genes [54, 55], detected colocalization of H2A.Z with WDR5 at *TFS1* in Col and decreased enrichment in *soc1-2* and *brm-1* (S9D–S9F Fig). In contrast, no difference in H2A.Z enrichment was detected between *spl* mutants and Col, further corroborating the idea that SPL functions to orchestrate transcriptional machinery rather than influencing nucleosomal composition (S9D–S9F Fig).

**Fig 7:**
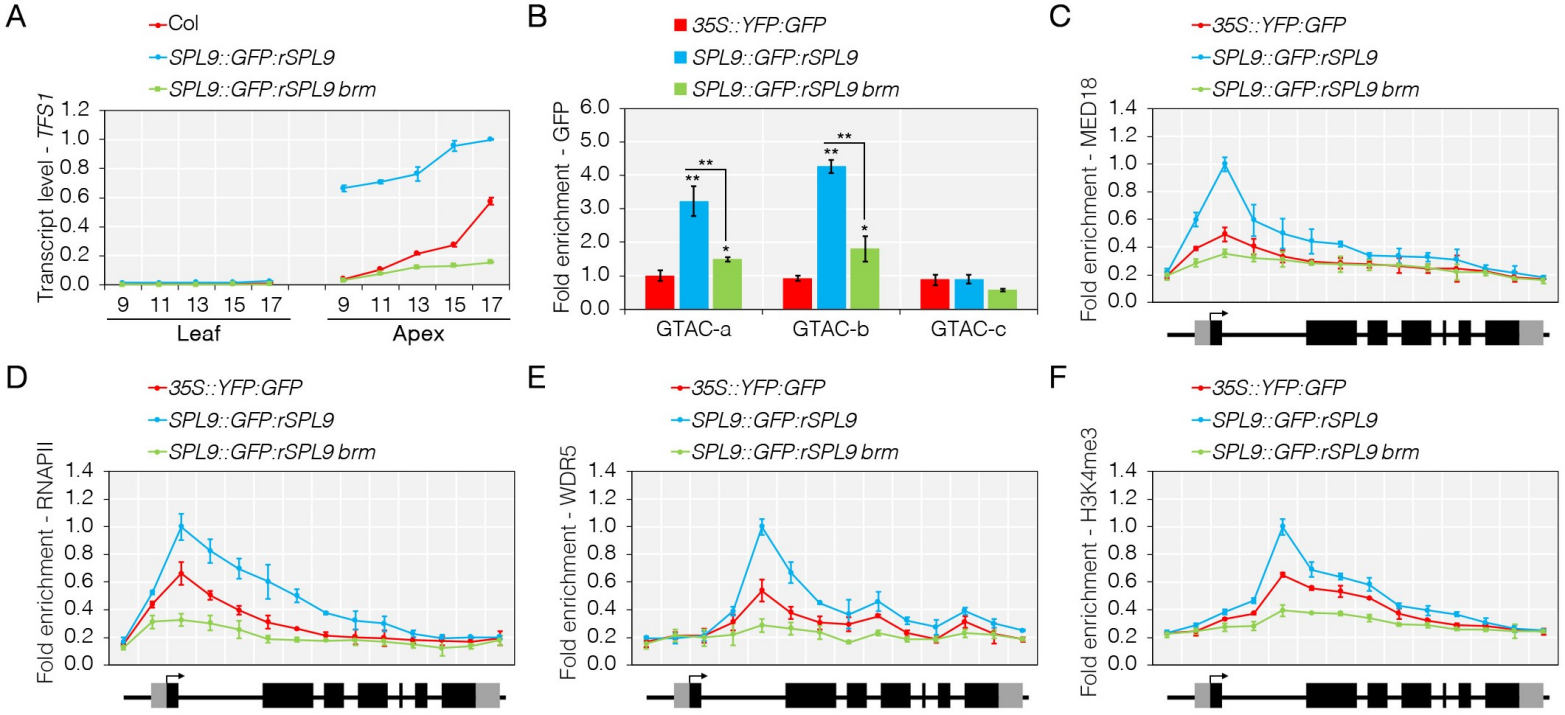
BRM is required for SPL9 dependent activation of *TFS1*. A) Elevated *TFS1* transcript levels in rSPL9 plants suppressed by *brm* mutation. Numbers at x-axis indicate number of long-days (LD) for which plants were grown prior to harvest. B) Binding of SPL9 at *TFS1* is reduced in *brm* mutants. Values were scaled to the value of amplicon GTAC-a in *35S*::*YFP*:*GFP*. Statistical significance was calculated against *35S*::*YFP*::*GFP* using Student’s *t*-test; *P < 0.05, **P < 0.01. C to F) SPL9-dependent recruitment of MED18 (C), RNAPII (D), WDR5 (E) and H3K4me3 (F) across gene body of *TFS1* is reduced in *SPL9*::*GFP*:*rSPL9 brm*.

The data described so far suggested that SOC1 mediated recruitment of BRM might enable association of SPL9 to chromatin. To test this idea, *SPL9*::*GFP*:*rSPL9* was introduced into *brm-1* mutants by genetic crossing. Unexpectedly, in *SPL9*::*GFP*:*rSPL9 brm-1* most of the floral structures were converted into carpelloid structures at the primary inflorescence, a more severe phenotype than either parental line (S10A and S10B Fig). To further characterize the molecular effect, *TFS1* transcript abundance was examined by RT-qPCR using RNA extracted from leaves and apices. The enhanced apex specific expression of *TFS1* in *SPL9*::*GFP*:*rSPL9* was strongly suppressed by *brm-1*, supporting the idea that BRM is required to support SPL9 activity (Fig 7A). Similarly, expression of other floral marker genes such as *SOC1*, *FUL*, *LEAFY* (*LFY*), *AP1* and *SEPALLATA3* (*SEP3*) was also reduced in this genotype, although SPL9 protein level was not affected (S10C–S10E Fig).

Consistent with the idea that BRM alters nucleosomal positioning leading to changes in the exposure of a critical SPL-binding site located at the 3’ end of *TFS1* (Fig 5I), reduced binding of GFP:rSPL9 was detected to the 5’ and 3’ end of *TFS1* in *brm-1* (Fig 7B). This result suggested that BRM facilitates binding of SPL9 to its cognate binding sites. SPL15 recruits RNAPII through Mediator [22], and consistent with SPL9 having a similar role at *TFS1*, a strong reduction in the recruitment of MED18, RNAPII and markers of active transcription such as WDR5 and H3K4me3 was detected in *brm-1* (Fig 7C–7F). Taken together, these data indicate that SOC1-dependent recruitment of BRM is required to allow SPL9 to bind to *TFS1* and that Mediator conveys regulatory information from SPL9 to the basal RNAPII transcriptional machinery that is coupled with the COMPASS-like complex to activate *TFS1* transcription.

## Discussion

The MADS box transcription factors FLC and SVP are well-established negative regulators of floral induction in Arabidopsis, however only fragmentary information is available on the roles of their direct targets in floral transition and the architecture of the regulatory network they control. Here, we addressed these issues by characterizing *TFS1*, an immediate target gene of FLC/SVP that encodes a B3-type transcription factor, which is expressed specifically on the flanks of the shoot apical meristem and promotes floral transition under LDs and SDs. *TFS1* transcription is repressed by FLC/SVP and promoted by SOC1, another MADS box transcription factor that is also encoded by a primary target of FLC/SVP. We show that FLC/SVP and SOC1 have opposing effects on transcription through mediating antagonistic histone modifications at *TFS1*. These data provide insight into the complexity of the regulatory network controlling floral transition downstream of FLC/SVP and define mechanisms by which MADS box transcription factors antagonistically regulate transcription of their direct targets.

### B3-Type transcription factors and plant reproductive development

TFS1 is a member of the B3-type transcription factor superfamily that is specific to the Viridiplantae [56]. Within this superfamily, TFS1 falls in the REM family, several of which have established or proposed roles in reproduction of Arabidopsis [30, 31]. Loss of function alleles of two members of this family, VERNALIZATION 1 (VRN1) and VERDANDI (VDD), provided genetic support for roles in reproductive development [57, 58]. VRN1 is required for stable transcriptional repression of *FLC* during induction of flowering by vernalization [57, 59], and appears to bind DNA non-specifically [57], while VDD is involved in ovule development [58]. In addition, several other members of this family are specifically expressed in the inflorescence meristem or developing flowers [31, 60–62]. REM transcription factors and MADS box proteins, another family of transcription factors with multiple roles in reproductive development, appear to often regulate one another’s expression. For example, VRN1 regulates *FLC*, *VDD* transcription is controlled by SEEDSTICK, *TFS1* is repressed by FLC/SVP and genome-wide studies of binding sites of MADS box factors AG, AP3, PI and AGL15 identified several REM genes as direct targets [31]. Both families of transcription factors are amplified in higher plants [30, 63], and they may have co-evolved to act in common pathways during the evolution of reproductive development.

The mechanism of action of REM proteins is not known, although they are believed to bind DNA via their B3 domains. A GFP-tagged form of VRN1 was found to associate widely with Arabidopsis chromosomes, and this association persisted through mitosis but was lost at meiosis [59]. Interestingly, TFS1 was also previously identified in a targeted proteomics approach as interacting with PCNA, a component of the DNA replication complex [64]. Also, our confocal imaging suggested that the nuclear localization and activity of TFS1 is closely related to cell division. The molecular functions of REM proteins such as TFS1 and how they are related to chromatin structure and cell division are interesting areas for future experimentation.

### Antagonistic effects of MADS box transcription factors on target gene expression

**Fig 8:**
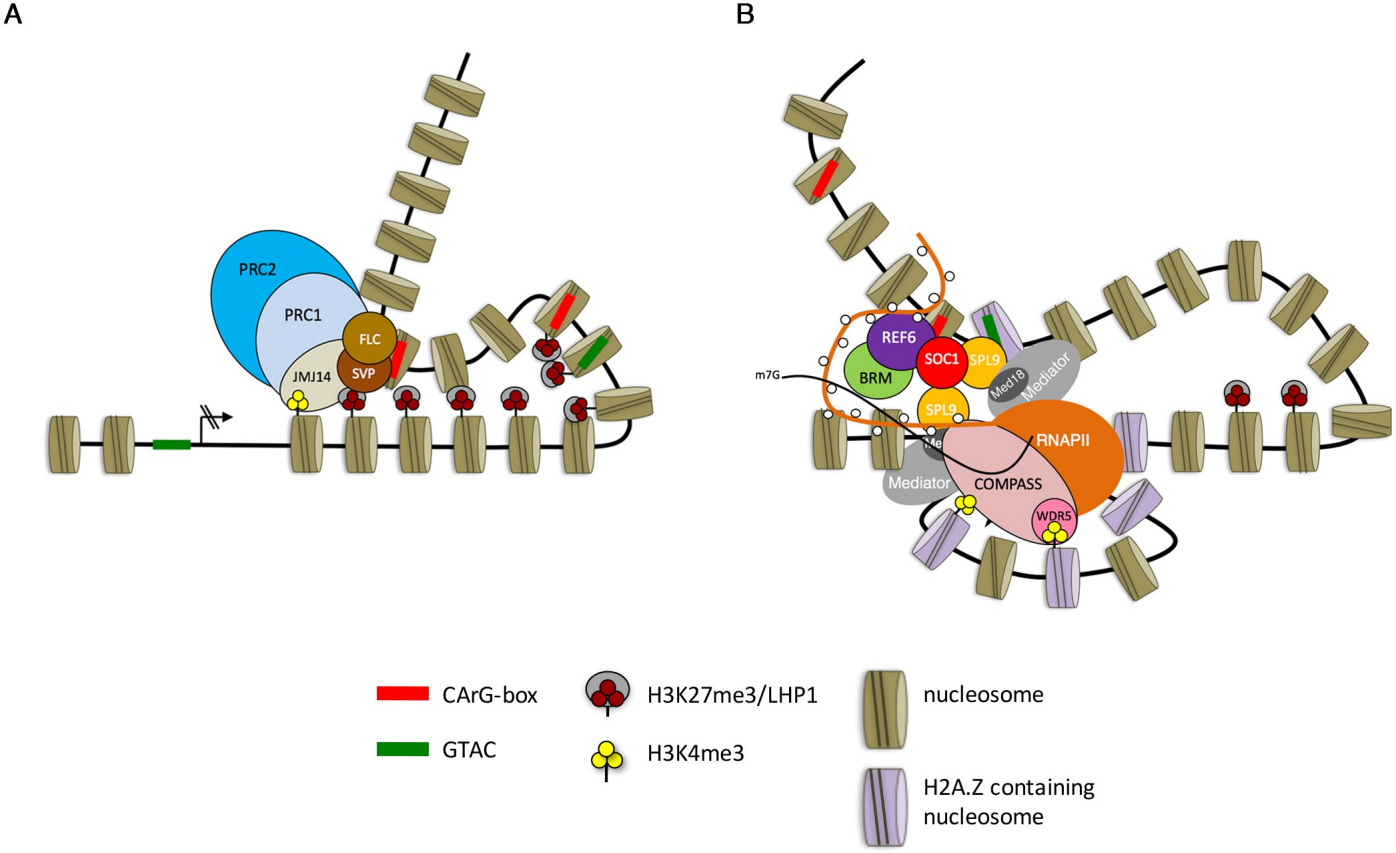
Proposed model for *TFS1* regulation and function by different flowering pathways. A) FLC and SVP mediated repression of *TFS1* requires PRC activity and a locked chromatin conformation. B) Activation of *TFS1* requires loop formation between 5’ and 3’ end and is mediated by the cooperativity between SOC1 and SPL9.

Genome-wide studies demonstrated that binding of FLC and SVP is predominately associated with transcriptional repression of target genes [6–8]. One of these targets is the flowering-time gene *FT*, whose expression in the vascular tissue of leaves is repressed by FLC and SVP [16, 65]. The capacity of FLC-like transcription factors to repress *FT* transcription has been reported to be associated with their ability to recruit PRC components and maintain H3K27me3 levels at the gene [19]. We also found that transcriptional repression of *TFS1* by FLC at the shoot meristem is associated with H3K27me3 accumulation, and that this involves formation of a chromatin loop between the 3’-end and intragenic regions of *TFS1* that requires PRC complexes. The JMJ14 H3K4 demethylase also associates with EMF1 and LHP1 [19–21], and we found that in *jmj14* mutants H3K27me3 levels as well as binding of both FLC and SVP were significantly reduced at *TFS1*, although the expression levels of *FLC* and *SVP* were not compromised. Collectively, these data suggest a model whereby PRC complexes involving EMF1, LHP1 and JMJ14 are recruited by FLC and SVP to *TFS1* to sustain H3K27me3 levels and binding of these transcription factors, thereby stably repressing *TFS1* transcription (Fig 8A). In this model, how the PRC1-like complexes reinforce binding of FLC and SVP and whether binding of these transcription factors is a prerequisite for PcG recruitment and PcG-mediated gene silencing remain to be resolved. Another possibility is that a co-factor for FLC binding, perhaps another MADS box transcription factor such as AGL16 [66], is reduced in expression in circumstances in which H3K27me3 levels are reduced. In this case, reduction in H3K27me3 would indirectly lower FLC binding.

In contrast to FLC/SVP, the MADS box factor SOC1 activates *TFS1* transcription during floral transition. Furthermore, SOC1 binds directly to *TFS1* as defined in genome-wide [12, 28] and targeted ChIP-qPCR experiments performed here. Induction of SOC1:GR was sufficient to activate *TFS1* transcription in the presence of SVP, demonstrating that SOC1 activation is epistatic to the repression mediated by SVP and that after SOC1:GR activation SVP binding to *TFS1* was strongly reduced. This reduction of SVP at *TFS1* could be due to its displacement by SOC1 binding to an adjacent CArG box or to the transcriptional repression of *SVP* by SOC1 [12, 15, 28]. Furthermore, our ChIP data indicate that the repressive state imposed by FLC/SVP is overcome by SOC1 through recruitment of the H3K27me3 demethylase REF6 and the chromatin remodeler BRM to the *TFS1* locus. Similarly, REF6 was recently shown to be recruited to targets by other MADS box transcription factors [67]. Our observations suggest that SOC1 displays characteristics associated with pioneer transcription factors, as it resolves condensed chromatin structures and opens chromatin through the combinatorial activity of REF6 and BRM. Similarly, a recent report in *Caenorhabditis elegans* demonstrated that the pioneer factor PHA-4 binds to promoters required for foregut development to recruit RNAPII and promote chromatin opening [68]. PHA-4 was proposed to facilitate chromatin opening by depositing RNAPII at target gene promoters. Similarly, the Drosophila maternal pioneer factor ZELDA (Zld) recruits poised RNAPII to Dorsal (Dl) target genes, facilitating chromatin accessibility for Dl which then mediates their zygotic activation [69, 70]. Accordingly, we found that the SOC1-REF6-BRM complex relaxes and opens chromatin at *TFS1* to facilitate binding of SPL9 and to activate poised RNAPII, resulting in a reduction in H3K27me3 levels across the *TFS1* genomic locus. Many genes directly repressed by FLC or SVP to maintain vegetative development are likely to be subsequently bound and activated by other MADS box transcription factors during reproductive development. Thus the mechanisms defined here by which SOC1 antagonises the repression of *TFS1* transcription imposed by FLC/SVP are likely to be more broadly relevant during the transition to flowering.

### Activating chromatin loop formed at *TFS1* through functional cooperation between SPL9 and SOC1

SOC1 functionally co-operates with SPL15 to form a chromatin loop associated with activation of *FUL* transcription [22]. Similarly, we showed by co-immunoprecipitation a physical interaction between SPL9 and SOC1 at *TFS1*. Similarly, we detected looping at the *TFS1* locus between the SPL9-binding region close to the TSS and the SOC1 binding region at the 3’-end of *TFS1* that might enable a higher turn-over rate of RNAPII to yield higher transcriptional activity. These observations suggest that the formation of an active chromatin loop could enable SOC1 and SPL9 to recruit respectively REF6 and RNAPII to the TSS, and then the active elongating RNAPII could cause the gene body of *TFS1* to change its position relative to the stable SOC1-SPL9 complex enabling REF6 to track along the gene with RNAPII progressively removing H3K27me3 (Fig 8B). This model predicts that the SPL9-SOC1 interaction induces dynamic chromatin folding that facilitates movement of the RNAPII along the gene body, rather than that RNAPII separates from the pre-initiation complex and tracks along the *TFS1* gene body. It will be interesting to determine in a genome-wide context whether other targets of SPL9 and SOC1 display similar features.

### TFS1 contributes to an inter-related network of interactions downstream of FLC

The analysis presented here incorporates *TFS1* into a network of interactions among FLC target genes. At the shoot meristem, FLC directly binds to and represses transcription of *SOC1* and *TFS1* [6, 7, 27, 43]. Furthermore, SOC1 directly activates the transcription of *TFS1*. Thus repression of *TFS1* by FLC involves both direct repression of expression of its positive activator SOC1 as well as direct repression of *TFS1*, a relationship characterized as a coherent feed forward loop type II [71]. The temporal and spatial patterns of *TFS1* expression on the flanks of the inflorescence meristem are overlapping with and partially conferred by SPL9, and may indicate an important role for *TFS1* in modulating the expression of genes in cells that will give rise to floral primordia. This suggestion is strengthened by the observation that the *Col-FRI flc-3 svp-41 tfs1*-1 triple mutant shows a floral morphology defect not shown by any of the single mutants. Previously, the *soc1-2 agl24-1 svp-41* combination was also demonstrated to have a synergistic effect on floral development due to redundancy among these transcription factors in the repression of genes involved in floral organ development [43]. Our data suggest that there may also be redundancy among FLC, SVP and TFS1 in the regulation of downstream genes, which could be characterized in a future analysis of TFS1 targets. More generally, our work emphasises that defining the network of genes negatively regulated by FLC/SVP, and understanding how these then interact during the progression to flowering when *FLC* expression is repressed or lost by mutation, is proving to be a productive approach in defining critical mechanisms controlling floral transition.

## Materials and methods

### Plant material and growth conditions

All seed stocks are in the Columbia-0 (Col-0) genetic background and were obtained from the Nottingham *Arabidopsis* Stock Centre (NASC; S1 Table) except for *35S*::*SOC1*:*GR soc1-1* (Hyun et al., 2016), which is in a Landsberg *erecta* (L*er*-0) genetic background. Seeds were sown on soil or on full-strength Murashige and Skoog (MS) medium containing 1% sucrose, stratified for 3 days at 4°C, and grown at 22°C under either long-days (16hrs light/8hrs dark; 150*μ*mol^.^m^-1.^s^-1^) or short-days (8hrs light/16hrs dark; 150*μ*mol^.^m^-1.^s^-1^). Plant age was measured when seeds were transferred from stratifying to ambient growth conditions.

### Plasmid construction and plant transformation

Full-length *TFS1* genomic region was cloned by PCR with Phusion Enzyme (New England Biolabs) according to the manufacturer’s recommendations and used to generate *TFS1*::*TFS1*::*9xAla-Venus*. To introduce 9xAla-Venus coding sequence, we employed Polymerase Incomplete Primer Extension (PIPE) cloning method [72] and plasmids were then introduced into *Agrobacterium* to transform Col plants by floral dip [73]. The sequences of the primers used for PIPE cloning are listed in S2 Table.

### RNA-extraction and real-time quantitative PCR

Total RNA of indicated genotypes at different days after sowing from leaves and apices was isolated with NucleoSpin RNA plant kit (Macherey-Nagel). DNA was removed by an on-column treatment with rDNase and 2 *μ*g RNA was reverse transcribed with an oligo(dT) primer, RNAseOUT Recombinant Ribonuclease Inhibitor (Thermo Fisher Scientific) and SuperScript II Reverse Transcriptase (Thermo Fisher Scientific). The cDNA equivalent of 20ng of total RNA was used in a 12 *μ*L qPCR reaction on a Roche Light Cycler 480 instrument (Roche) with either iQ SYBR Green Supermix (BioRad) or GoTaq qPCR Master Mix (Promega) and quantified using the *UBC21* (AT5G25760) as a reference gene to which data was normalized [74]. The mean of three biological replicates with standard deviation is shown and list of primers used for expression analyses can be found in S2 Table.

### Chromatin-immunoprecipitation (ChIP) assays

ChIP was performed as previously described with minor modifications [22]. In brief, above-ground tissue of 15LD-grown plants was collected at ZT8 and fixed in PBS solution containing 1.5% formaldehyde. ChIP-assays in which indirect binding of the protein of interest to chromatin was studied, Di(N-succinimidyl) glutarate (DSG; Synchem) at a final concentration of 1 *μ*M was used to introduce protein-protein crosslinks prior to formaldehyde-assisted protein-chromatin crosslinking. To determine fold enrichment levels, ChIP-DNA was quantified on a Roche Light Cycler 480 instrument (Roche) with iQ SYBR Green Supermix (BioRad) and normalized against *ACT8* (AT1G49240). In ChIP assays in which histone modifications were tested, the values of the tested histone marks were normalized against histone H3. The average of three biological replicates is shown and list of primers used for fold enrichment analyses can be found in S2 Table.

### Chromatin conformation capture (3C) assay

3C assay was performed as described previously with minor modifications. A total of 2g of above-ground tissue of 15 day-old LD-grown plants was used for 3C study. Chromatin DNA was digested for 16 hrs at 37°C with 400U Sau3AI (New England Biolabs, S3 Table) while agitating at 900 r.p.m. For intramolecular ligation, digested nuclei were incubated for 5 hrs at 16°C in 500 U T4 DNA Ligase (Promega, S3 Table). In parallel, the cloned TFS1:9xAla-Venus construct was digested and ligated. The 3C DNA ligation products were quantified by RT-qPCR and normalised to the TFS1:9xAla-Venus control using the delta-delta Ct method. The sequences of the primers used in the 3C-assay are listed in S2 Table.

### Micrococcal nuclease-assay (MNase-assay)

Micrococcal nuclease-assay was performed as described previously with minor modifications [75]. For nuclear extraction, above-ground tissue of 15 day-old LD-grown plants was harvested, ground in liquid nitrogen and resuspended in lysis buffer (LB) [50 mM HEPES pH7.5, 150 mM NaCl, 1 mM EDTA, 1% Triton X-100, 10% glycerol, 5 mM ß-mercaptoethanol and protease inhibitor cocktail (Roche)]. After 1 hr of lysis, the lysis mixture was filtered twice through 2 layers of Miracloth (Calbiochem) and protocol was followed as previously described. For MNase treatment, nuclei were treated with 5U MNase (Thermo Fisher Scientific) for 15 min and digest was stopped by adding 16 μL 250 mM EDTA, and then treated with RNase A and Proteinase K (Sigma Aldrich), each for 1 hr.

### Protein analyses

Total protein extraction and *in vivo* co-immunoprecipitation were performed as described previously with minor modifications [22, 76]. For SVP Western-analysis, roughly 50 apices of 15 day-old LD-grown plants were harvested. Protein concentration was determined by Bradford series and a total of 50μg for crude extract and 1mg for immunoprecipitation was used. The amino acid sequences of the epitopes for generating SVP antibody are presented in S4 Table.

### *In*-*situ* hybridisation and imaging

*In-situ* hybridisation was performed according to the method described previously [77]. The sequences of the primers used for the *in-situ* hybridisation experiments are listed in S2 Table. For confocal microscopy, shoot apices at different developmental stages were collected and fixed with 4% paraformaldehyde (PFA) prepared in phosphate-buffered saline (PBS) at pH7.0. Samples were then vacuum infiltrated for 20 min on ice, transferred to fresh 4% PFA, and stored at 4°C overnight. The fixed samples were washed twice for 1 min in PBS, then cleared with ClearSee [10% (w/v) xylitol, 15% (w/v) sodium deoxycholate and 25% (w/v) urea][78] for 3 to 8 days at room temperature. After clearing, the shoot meristems were imaged by confocal laser scanning microscopy (Zeiss LSM780), as described previously [79].

### Image processing and figure construction

All image processing and figure construction was performed with Photoshop (www.adobe.com).

### Accession numbers

Mutant and transgenic lines used in this study, including references for their origin and description in literature, and their respective AGI identifiers are listed in S1 Table.

## Supporting information

S1 Fig Roles of *TFS1* and *TFS1-like* genes in flowering. A) Phylogenetic tree for B3 type gene family based on ClustalW alignment with a bootstrap value of 1.000 replicates. B) Immunoblot to test for cross-reactivity of SVP. Triangle indicates specific whereas asterisks indicate non-specific bands. Values on the left of the Western-blots indicated molecular weight (kDa). C) Transcript level of *TFS1*. Plants were grown for 2 weeks in short-days and then transferred to long-day. Values were scaled to set value of apices of 2 weeks short-day grown *Col-FRI* plants to 1. D) Increased expression of *TFS1* in apices of *Col-FRI* plants when transferred to ambient growth condition after vernalisation. NV, non-vernalised; V, weeks vernalised in short-days (SD). E) Spatial pattern of expression of *TFS1* assessed by *in-situ* hybridization after floral transition. DAS, days after sowing.
(TIF)

S2 Fig Genetic analysis of role of *TFS1* in flowering. A to D) Comparison of *tfs1* mutant plants and Columbia grown in either LDs (A) or in SDs (C). Leaf number of *tfs1* plants compared to Columbia grown in LDs (B) and in SDs (D). Statistical significance was calculated using Student’s *t*-test; **P < 0.01. E) Representative gene model for *TFS1* and T-DNA insertion used in this study. F) Total leaf number of *tfs1-1* and *tfs1-2* plants compared to Col. Statistical significance was calculated using Student’s *t*-test; *P < 0.05. G) Inflorescence of *Col-FRI flc svp tfs1* plants. H) Floral structures of *Col-FR flc svp tfs1* plants. Particular features are marked: blue arrow, misplaced floral organs; white arrow, ectopic ovules; yellow arrow, stigmatic papillae on leaf-like structures; red arrow, leaf-like anthers.
(TIF)

S3 Fig Responsiveness of *TFS1* transcript abundance to vernalisation. A) H3K27me3 levels are increased at TTS of *TFS1* in *Col-FRI flc svp*. B and C) H3K4me3 levels are increased in *Col-FRI flc svp* (B) and *Col-FRI clf* and *Col-FRI jmj14* (C) plants. Values were scaled to set highest value in *Col-FRI flc svp* to 1. D) Reduced *TFS1* transcript levels in *Col-FRI* plants suppressed by *clf* mutation. E and F) Transcript levels of *FLC* (E) and *SVP* (F) in leaves and apices of *Col-FRI clf* plants. Numbers at x-axis indicate number of long-days (LD) for which plants were grown prior to harvest. G) Reduced *TFS1* transcript levels in *Col-FRI* plants suppressed by *jmj14* mutation. H and I) Transcript levels of *FLC* (H) and *SVP* (I) in leaves and apices of *Col-FRI jmj14* plants. Numbers at x-axis indicate number of long-days (LD) for which plants were grown prior to harvest. Statistical significance was calculated using Student’s *t*-test; *P < 0.05, **P < 0.01, n.s. *P* > 0.05.
(TIF)

S4 Fig SOC1-induced removal of H3K27me3 and transcriptional activation of *TFS1*. A) SOC1:GR-induced transcriptional activation of *TFS1*. B and C) Top: ChIP-qPCR analysis of SOC1 (B) and SVP (C) on CArG-boxes at *TFS1* in *35S*::*SOC1*:*GR soc1* plants after DEX treatment. Bottom: Diagram of *TFS1* locus with CArG-boxes and amplicons used for ChIP-qPCR. Red and orange triangles indicate CArG-boxes in *TFS1*. Green and red lines represent amplicons used for ChIP-qPCR study. D) ChIP-qPCR analysis of H3K27me3 at *TFS1* in *35S*::*SOC1*:*GR soc1* plants after DEX treatment. B to D, values were scaled to set value of first primers at 20min after DEX treatment in *35S*::*SOC1*:*GR soc1* to 1. GB, indicates *G*ene *B*ody amplicon used for ChIP-qPCR. Statistical significance was calculated against mock treatment using Student’s *t*-test; *P < 0.01, n.s. *P* > 0.05. Representative confocal images of independent transformants of E) TFS1:9AV, F) TFS1:9AV mCArGII and G) TFS1:9AV mCArGI+II. H) Abundance of *TFS1* mRNA in indicated transgenic plants as well as in Col and *tfs1* controls.
(TIF)

S5 Fig Flowering time analysis of *TFS1*:*9AV* transgenic lines. A and B) Representative photograph (A) and total leaf number (B) of *TFS1*::*TFS1*:*9AV* in *tfs1*. C and D) Representative photograph (C) and total leaf number (D) of *TFS1*::*TFS1*:*9AV mCArGII* in *tfs1*. E and F) Representative photograph (E) and total leaf number (F) of *TFS1*::*TFS1*:*9AV mCArGII* in *soc1*. G and H) Representative photograph (G) and total leaf number (H) of *TFS1*::*TFS1*:*9AV mCArGI+II* in *tfs1*. In each case, transformants are shown in the same order in the representative photograph as in the leaf number plot. Statistical significance was calculated using Student’s *t*-test; *P < 0.1, **P < 0.05, ***P < 0.01, n.s. *P* > 0.05.
(TIF)

S6 Fig *TFS1* is expressed at the periphery of the apical meristem. A) Top view of *TFS1*:*9xAla-Venus* (yellow) expression at the apical meristem. DAPI (blue) serves to highlight individual cells. B) Side view of TFS1:9xAla-Venus (yellow) localisation at the shoot apical meristem. C to G) TFS1:9xAla-Venus localises to the nucleus of sepal cells (C), in young flowers and at the base of pedicels (D) and in flowers of stage stage 3 (E), stage 2 (F) and stage 4 (G) after LMB treatment.
(TIF)

S7 Fig SPL9 and SOC1 cooperatively activate *TFS1*. A) ChIP-qPCR for rSPL9 binding to putative SPL-binding sites at *TFS1* locus. Statistical significance was calculated against the respective amplicon of 35S::YFP:GFP. B) ChIP-qPCR for temporal binding of SPL9:GR at *TFS1* after DEX treatment. Statistical significance was calculated against the 20min DEX treatment. C to E) ChIP-qPCR for MED18 (C), RNAPII (D) and H3K4me3 (E) at *TFS1* locus. Plants were grown for 15 LD and harvested at ZT8. F) Spatial pattern of expression of *TFS1* assessed by *in-situ* hybridization during floral transition in Col and *GFP*:*rSPL9*. Plants were grown for 2 weeks in SD and then transferred to permissive LD. G) Confocal images of *TFS1*:*9AV mGTACa1/a2* mutant transgenic lines. Statistical significance was calculated using Student’s *t*-test; *P < 0.05, **P < 0.01, n.s. *P* > 0.05.
(TIF)

S8 Fig SPL activity is required for *TFS1* expression. A and B) *TFS1* transcript abundance in short-days is increased by SPL and SOC1 (A) and REF6 (B). Data are shown for 3–8 weeks after germination and values were scaled to set value of 3 weeks SD-grown Col plants to 1. C) Reduced expression of *TFS1* in *ga1* under LD conditions. D) Spatio-temporal expression pattern of *TFS1* assessed by *in situ* hybridization in LD. E) *TFS1* mRNA levels are reduced in apices of *spl* mutants during floral transition. Values were scaled to set value of 7 LD of Col to 1. F to H) Reduced RNAPII (F) and H3K4me3 (G) and increased H3K27me3 (H) levels at *TFS1* in *spl* mutants. Values were scaled to highest value in Col (F and G) and *spl9 spl15* (H) to 1. In A to H, Plants were harvested at ZT8.
(TIF)

S9 Fig WDR5 and H2A.Z levels at *TFS1* depend on SPL and BRM activity, respectively. A to C) WDR5 levels at *TFS1* depend on SOC1 (A), BRM (B) and SPL (C). D to F) H2A.Z incorporation at *TFS1* depends on SOC1 (D) and BRM (E) but not on SPL (F). ChIP-PCR was performed on 15 LD grown plants that were harvested at ZT8 (A to F).
(TIF)

S10 Fig Phenotypes of *rSPL9 brm* plants and marker gene expression analyses. A and B) 5-weeks old long-day grown *GFP*:*rSPL9 brm* plants display altered plant architecture (A) and partial transformation of flowers into carpelloid structures (B). C) GFP Western analysis to detect GFP:SPL9 in Col and *brm*. Values on the left of the Western-blots indicated molecular weight (kDa). D and E) Comparative expression analysis of marker genes expressed in leaves (D) and apices (E) of Col, *GFP*:*rSPL9* and *GFP*:*rSPL9 brm* plants. X-axis indicates number of long-days (LD) for which plants were grown prior to harvest.
(TIF)

S1 Table Mutant and transgenic lines used in this study. (PDF)

S2 Table List of oligonucleotides used in this study. (PDF)

S3 Table List of antibodies and additional consumables used in this study. (PDF)

S4 Table List of position and amino acid sequence of epitope to generate SVP antibody. (PDF)

S5 Table List of the flowering time of various mutants and tagged lines used in this study. (PDF)
