# Supplementary figures and images for: Floral regulators FLC and SOC1 directly regulate expression of the B3-type transcription factor TARGET OF FLC AND SVP 1 at the Arabidopsis shoot apex via antagonistic chromatin modifications
Source: PLoS Genet. 2019 Apr 4;15(4):e1008065. doi: 10.1371/journal.pgen.1008065 (PMC6467423; doi:10.1371/journal.pgen.1008065)

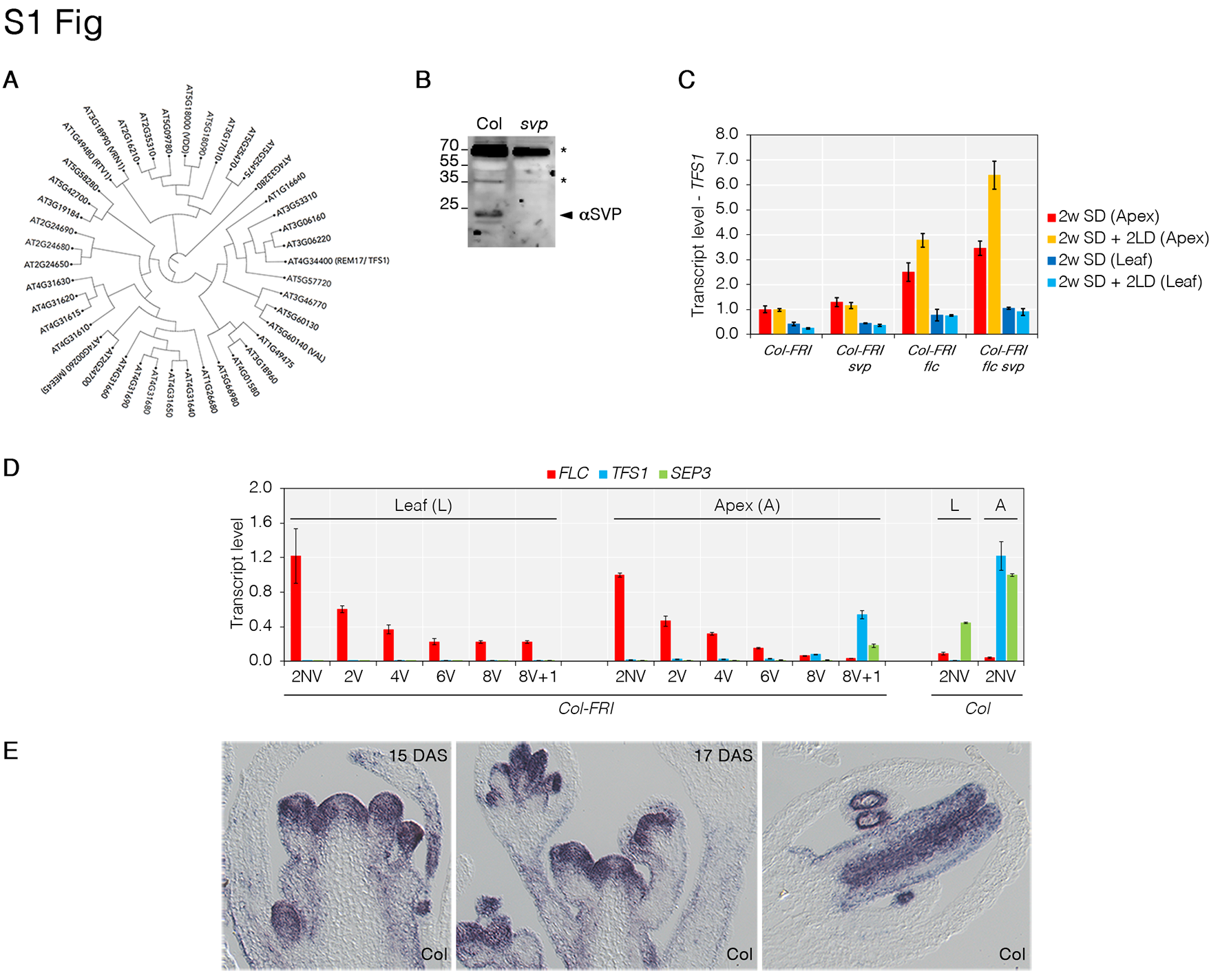

Supplement: S1 Fig — A) Phylogenetic tree for B3 type gene family based on ClustalW alignment with a bootstrap value of 1.000 replicates. B) Immunoblot to test for cross-reactivity of SVP. Triangle indicates specific whereas asterisks indicate non-specific bands. Values on the left of the Western-blots indicated molecular weight (kDa). C) Transcript level of TFS1. Plants were grown for 2 weeks in short-days and then transferred to long-day. Values were scaled to set value of apices of 2 weeks short-day grown Col-FRI plants to 1. D) Increased expression of TFS1 in apices of Col-FRI plants when transferred to ambient growth condition after vernalisation. NV, non-vernalised; V, weeks vernalised in short-days (SD). E) Spatial pattern of expression of TFS1 assessed by in-situ hybridization after floral transition. DAS, days after sowing. (TIF) [file pgen.1008065.s001.tif]

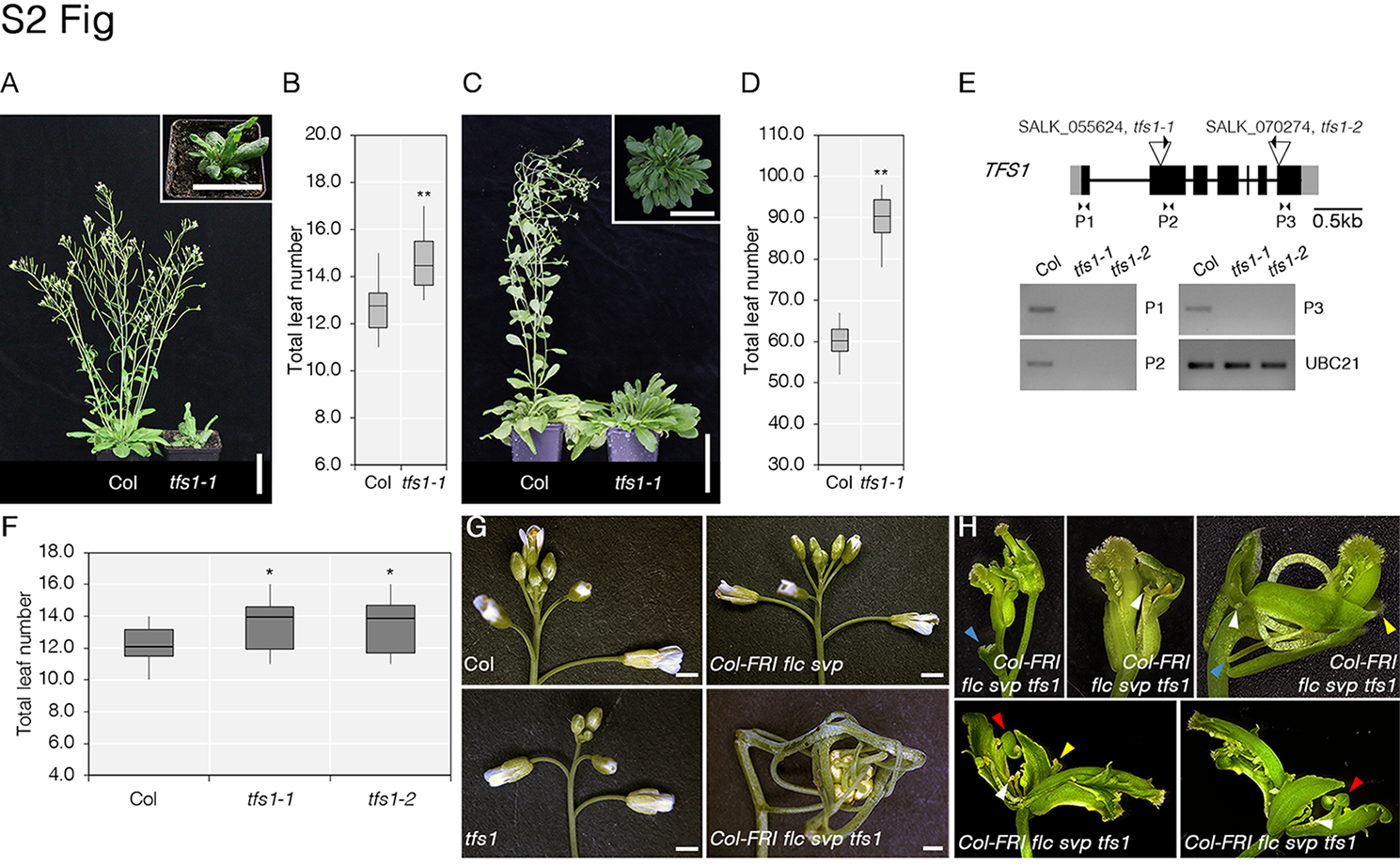

Supplement: S2 Fig — A to D) Comparison of tfs1 mutant plants and Columbia grown in either LDs (A) or in SDs (C). Leaf number of tfs1 plants compared to Columbia grown in LDs (B) and in SDs (D). Statistical significance was calculated using Student’s t-test; **P < 0.01. E) Representative gene model for TFS1 and T-DNA insertion used in this study. F) Total leaf number of tfs1-1 and tfs1-2 plants compared to Col. Statistical significance was calculated using Student’s t-test; *P < 0.05. G) Inflorescence of Col-FRI flc svp tfs1 plants. H) Floral structures of Col-FR flc svp tfs1 plants. Particular features are marked: blue arrow, misplaced floral organs; white arrow, ectopic ovules; yellow arrow, stigmatic papillae on leaf-like structures; red arrow, leaf-like anthers. (TIF) [file pgen.1008065.s002.tif]

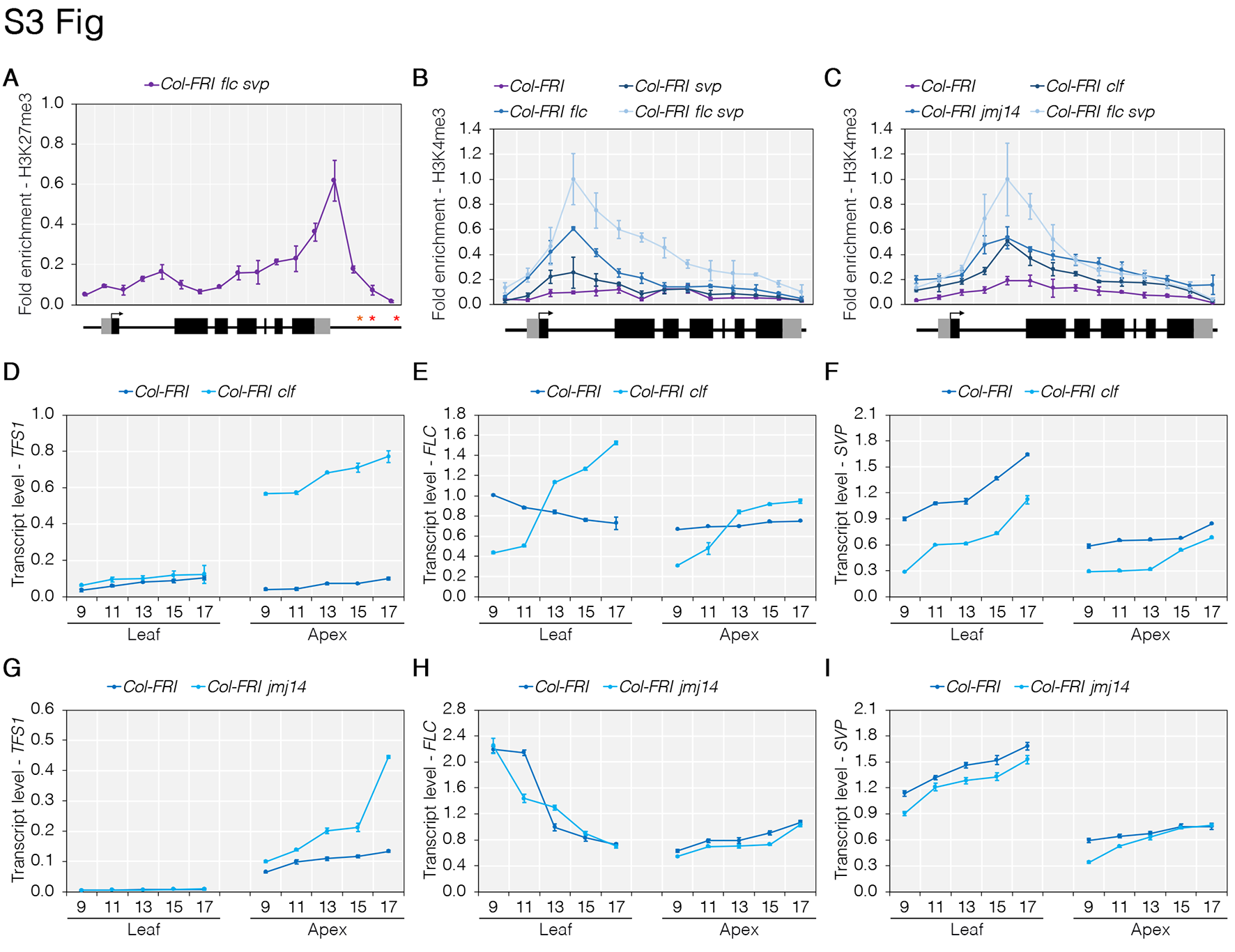

Supplement: S3 Fig — A) H3K27me3 levels are increased at TTS of TFS1 in Col-FRI flc svp. B and C) H3K4me3 levels are increased in Col-FRI flc svp (B) and Col-FRI clf and Col-FRI jmj14 (C) plants. Values were scaled to set highest value in Col-FRI flc svp to 1. D) Reduced TFS1 transcript levels in Col-FRI plants suppressed by clf mutation. E and F) Transcript levels of FLC (E) and SVP (F) in leaves and apices of Col-FRI clf plants. Numbers at x-axis indicate number of long-days (LD) for which plants were grown prior to harvest. G) Reduced TFS1 transcript levels in Col-FRI plants suppressed by jmj14 mutation. H and I) Transcript levels of FLC (H) and SVP (I) in leaves and apices of Col-FRI jmj14 plants. Numbers at x-axis indicate number of long-days (LD) for which plants were grown prior to harvest. Statistical significance was calculated using Student’s t-test; *P < 0.05, **P < 0.01, n.s. P > 0.05. (TIF) [file pgen.1008065.s003.tif]

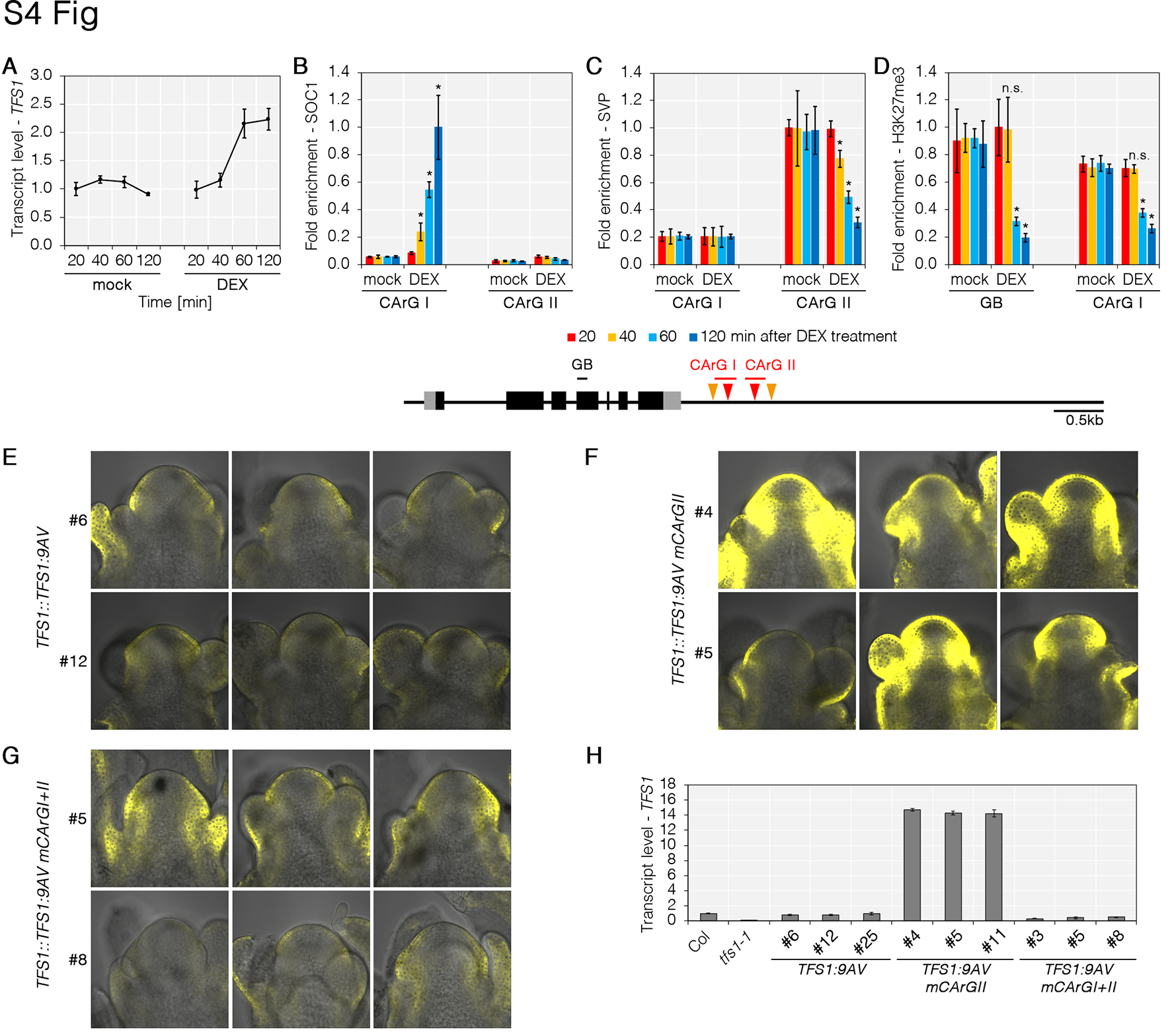

Supplement: S4 Fig — A) SOC1:GR-induced transcriptional activation of TFS1. B and C) Top: ChIP-qPCR analysis of SOC1 (B) and SVP (C) on CArG-boxes at TFS1 in 35S::SOC1:GR soc1 plants after DEX treatment. Bottom: Diagram of TFS1 locus with CArG-boxes and amplicons used for ChIP-qPCR. Red and orange triangles indicate CArG-boxes in TFS1. Green and red lines represent amplicons used for ChIP-qPCR study. D) ChIP-qPCR analysis of H3K27me3 at TFS1 in 35S::SOC1:GR soc1 plants after DEX treatment. B to D, values were scaled to set value of first primers at 20min after DEX treatment in 35S::SOC1:GR soc1 to 1. GB, indicates Gene Body amplicon used for ChIP-qPCR. Statistical significance was calculated against mock treatment using Student’s t-test; *P < 0.01, n.s. P > 0.05. Representative confocal images of independent transformants of E) TFS1:9AV, F) TFS1:9AV mCArGII and G) TFS1:9AV mCArGI+II. H) Abundance of TFS1 mRNA in indicated transgenic plants as well as in Col and tfs1 controls. (TIF) [file pgen.1008065.s004.tif]

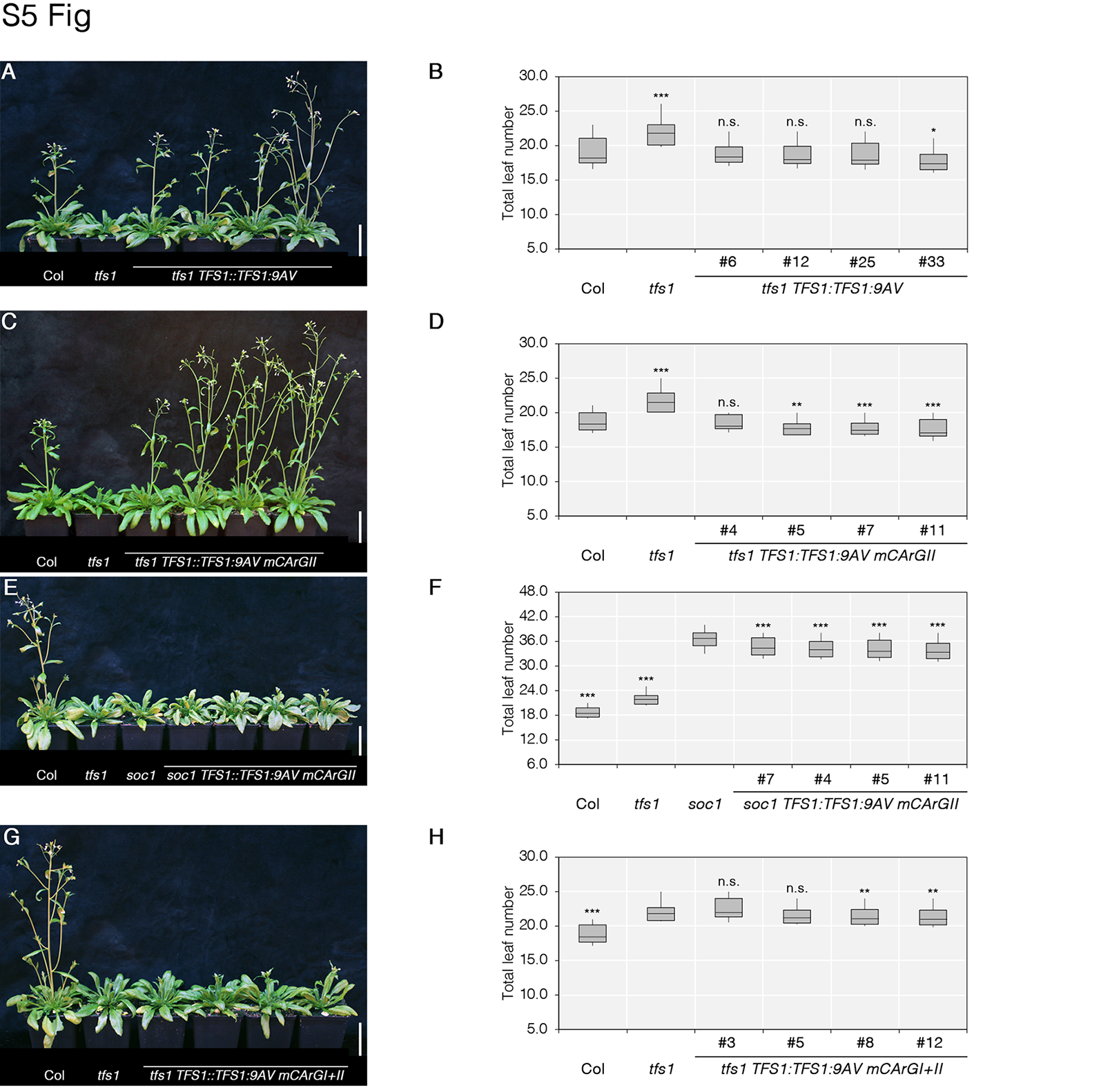

Supplement: S5 Fig — A and B) Representative photograph (A) and total leaf number (B) of TFS1::TFS1:9AV in tfs1. C and D) Representative photograph (C) and total leaf number (D) of TFS1::TFS1:9AV mCArGII in tfs1. E and F) Representative photograph (E) and total leaf number (F) of TFS1::TFS1:9AV mCArGII in soc1. G and H) Representative photograph (G) and total leaf number (H) of TFS1::TFS1:9AV mCArGI+II in tfs1. In each case, transformants are shown in the same order in the representative photograph as in the leaf number plot. Statistical significance was calculated using Student’s t-test; *P < 0.1, **P < 0.05, ***P < 0.01, n.s. P > 0.05. (TIF) [file pgen.1008065.s005.tif]

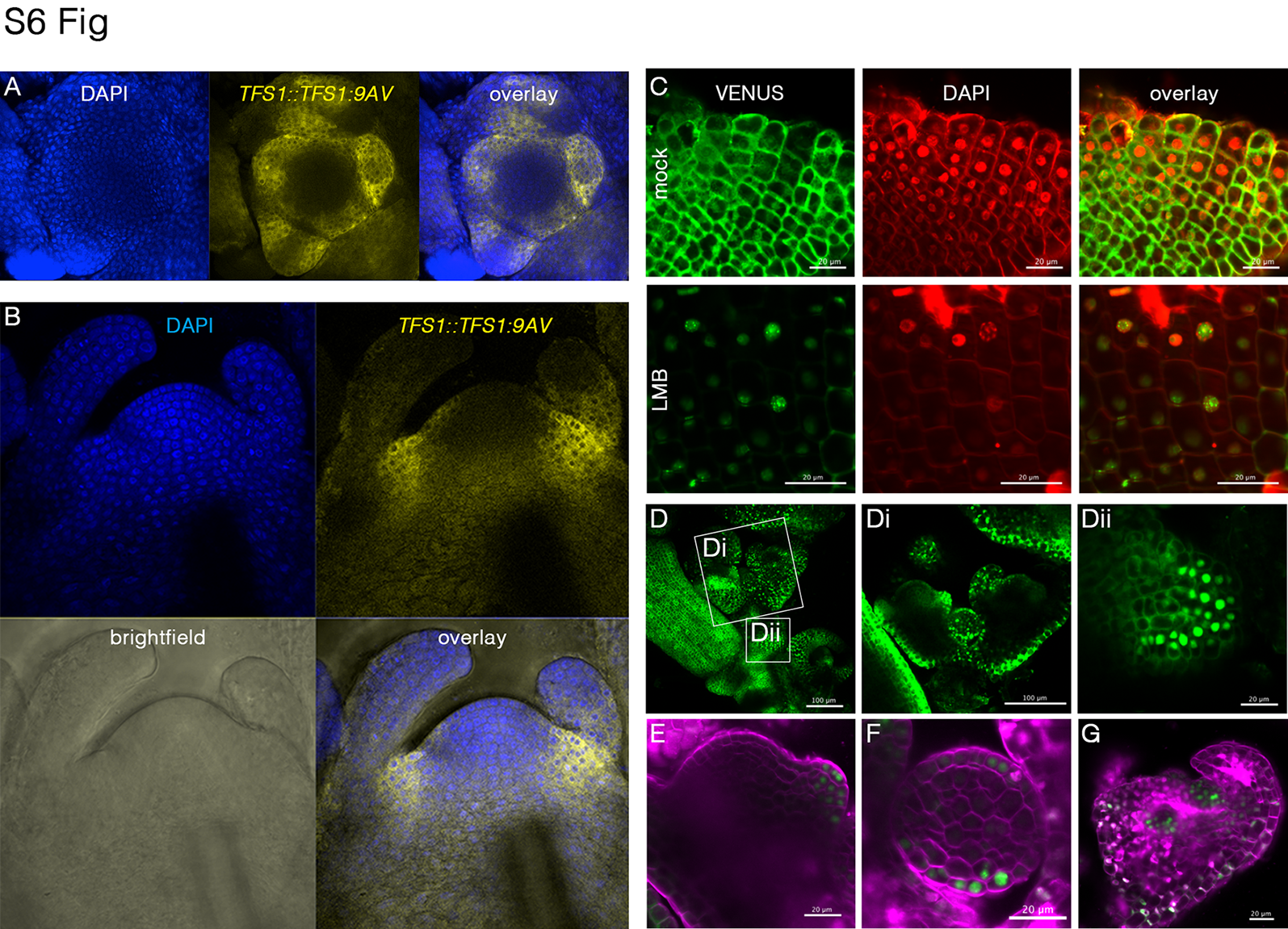

Supplement: S6 Fig — A) Top view of TFS1:9xAla-Venus (yellow) expression at the apical meristem. DAPI (blue) serves to highlight individual cells. B) Side view of TFS1:9xAla-Venus (yellow) localisation at the shoot apical meristem. C to G) TFS1:9xAla-Venus localises to the nucleus of sepal cells (C), in young flowers and at the base of pedicels (D) and in flowers of stage stage 3 (E), stage 2 (F) and stage 4 (G) after LMB treatment. (TIF) [file pgen.1008065.s006.tif]

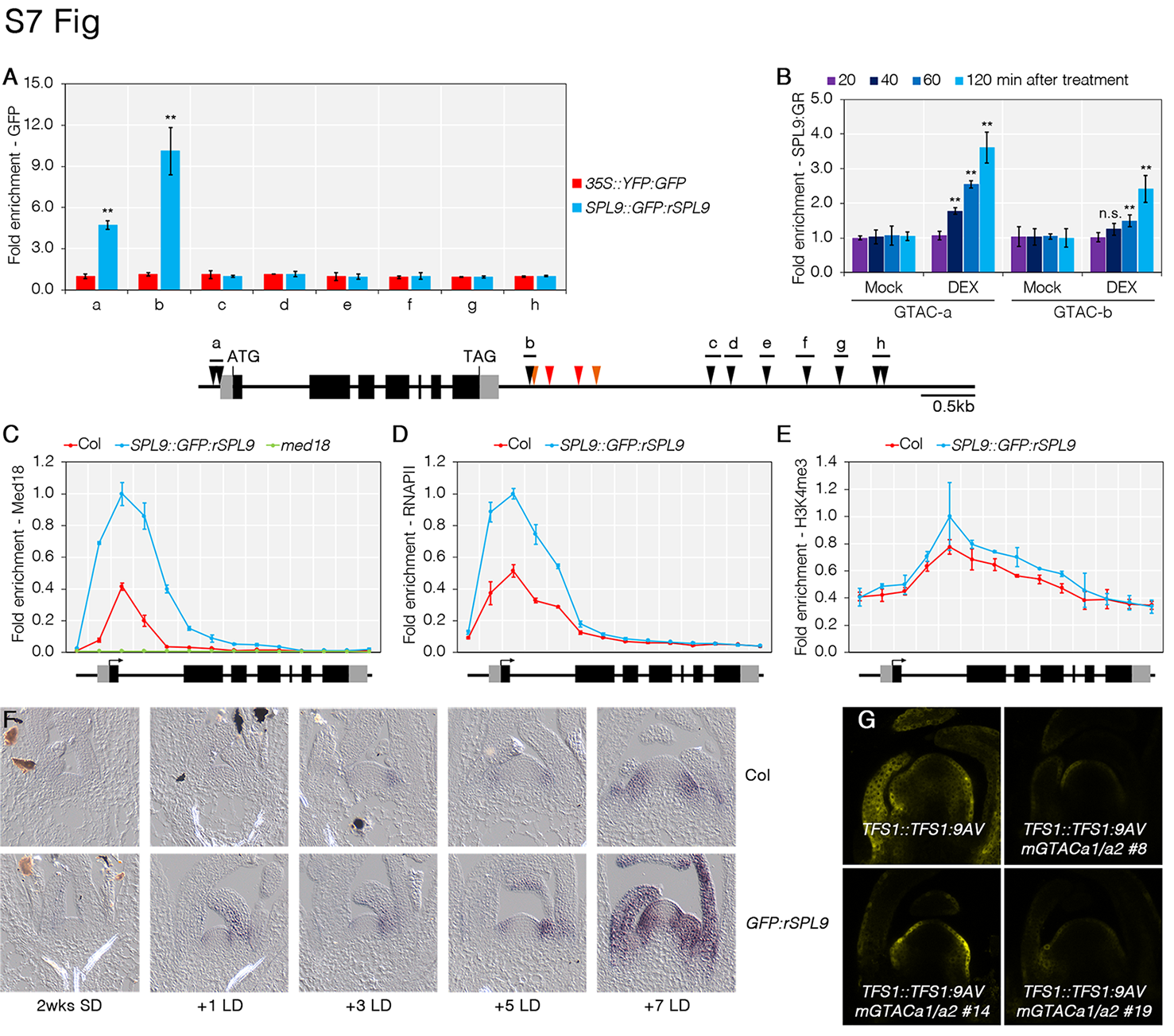

Supplement: S7 Fig — A) ChIP-qPCR for rSPL9 binding to putative SPL-binding sites at TFS1 locus. Statistical significance was calculated against the respective amplicon of 35S::YFP:GFP. B) ChIP-qPCR for temporal binding of SPL9:GR at TFS1 after DEX treatment. Statistical significance was calculated against the 20min DEX treatment. C to E) ChIP-qPCR for MED18 (C), RNAPII (D) and H3K4me3 (E) at TFS1 locus. Plants were grown for 15 LD and harvested at ZT8. F) Spatial pattern of expression of TFS1 assessed by in-situ hybridization during floral transition in Col and GFP:rSPL9. Plants were grown for 2 weeks in SD and then transferred to permissive LD. G) Confocal images of TFS1:9AV mGTACa1/a2 mutant transgenic lines. Statistical significance was calculated using Student’s t-test; *P < 0.05, **P < 0.01, n.s. P > 0.05. (TIF) [file pgen.1008065.s007.tif]

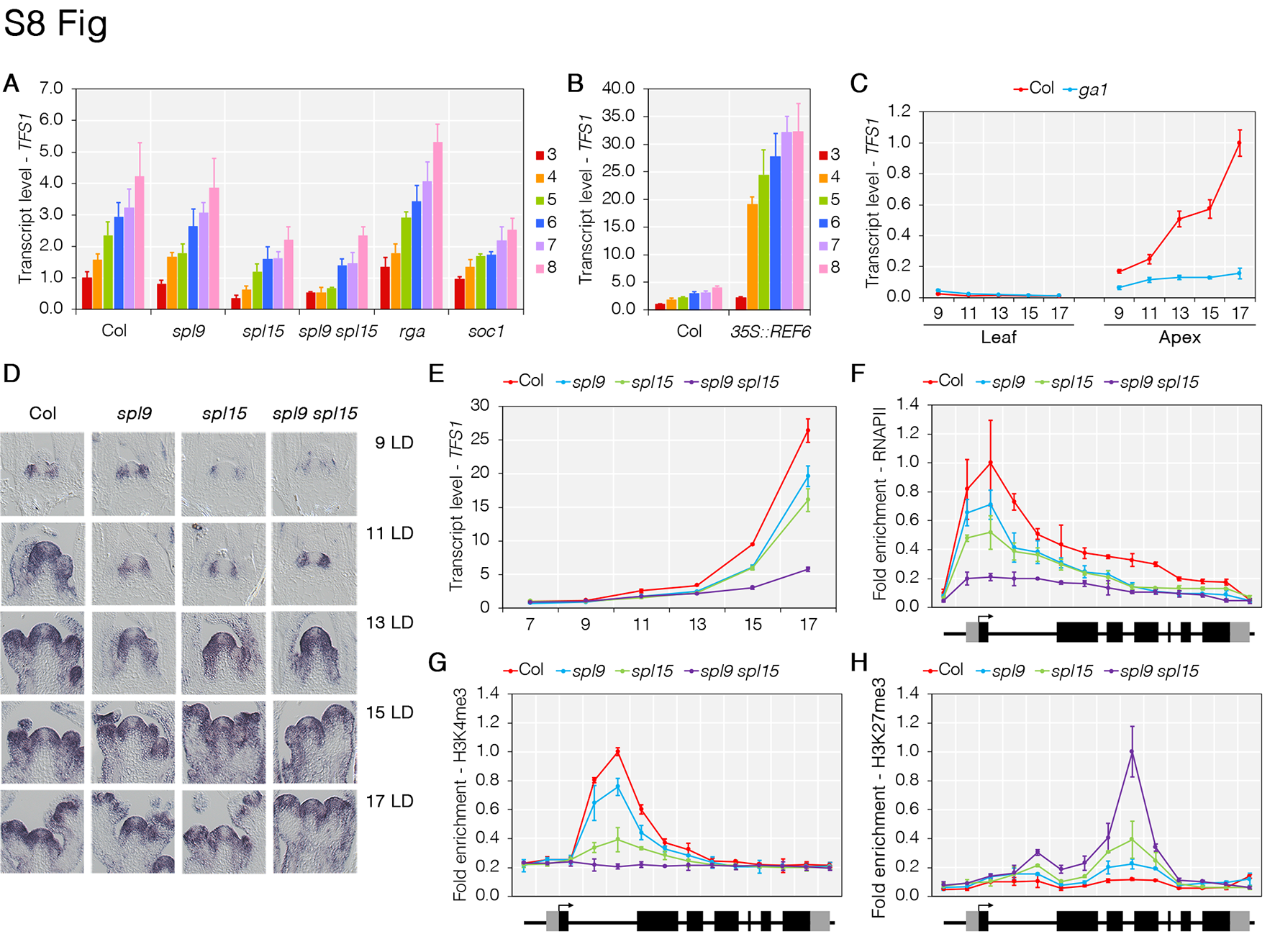

Supplement: S8 Fig — A and B) TFS1 transcript abundance in short-days is increased by SPL and SOC1 (A) and REF6 (B). Data are shown for 3–8 weeks after germination and values were scaled to set value of 3 weeks SD-grown Col plants to 1. C) Reduced expression of TFS1 in ga1 under LD conditions. D) Spatio-temporal expression pattern of TFS1 assessed by in situ hybridization in LD. E) TFS1 mRNA levels are reduced in apices of spl mutants during floral transition. Values were scaled to set value of 7 LD of Col to 1. F to H) Reduced RNAPII (F) and H3K4me3 (G) and increased H3K27me3 (H) levels at TFS1 in spl mutants. Values were scaled to highest value in Col (F and G) and spl9 spl15 (H) to 1. In A to H, Plants were harvested at ZT8. (TIF) [file pgen.1008065.s008.tif]

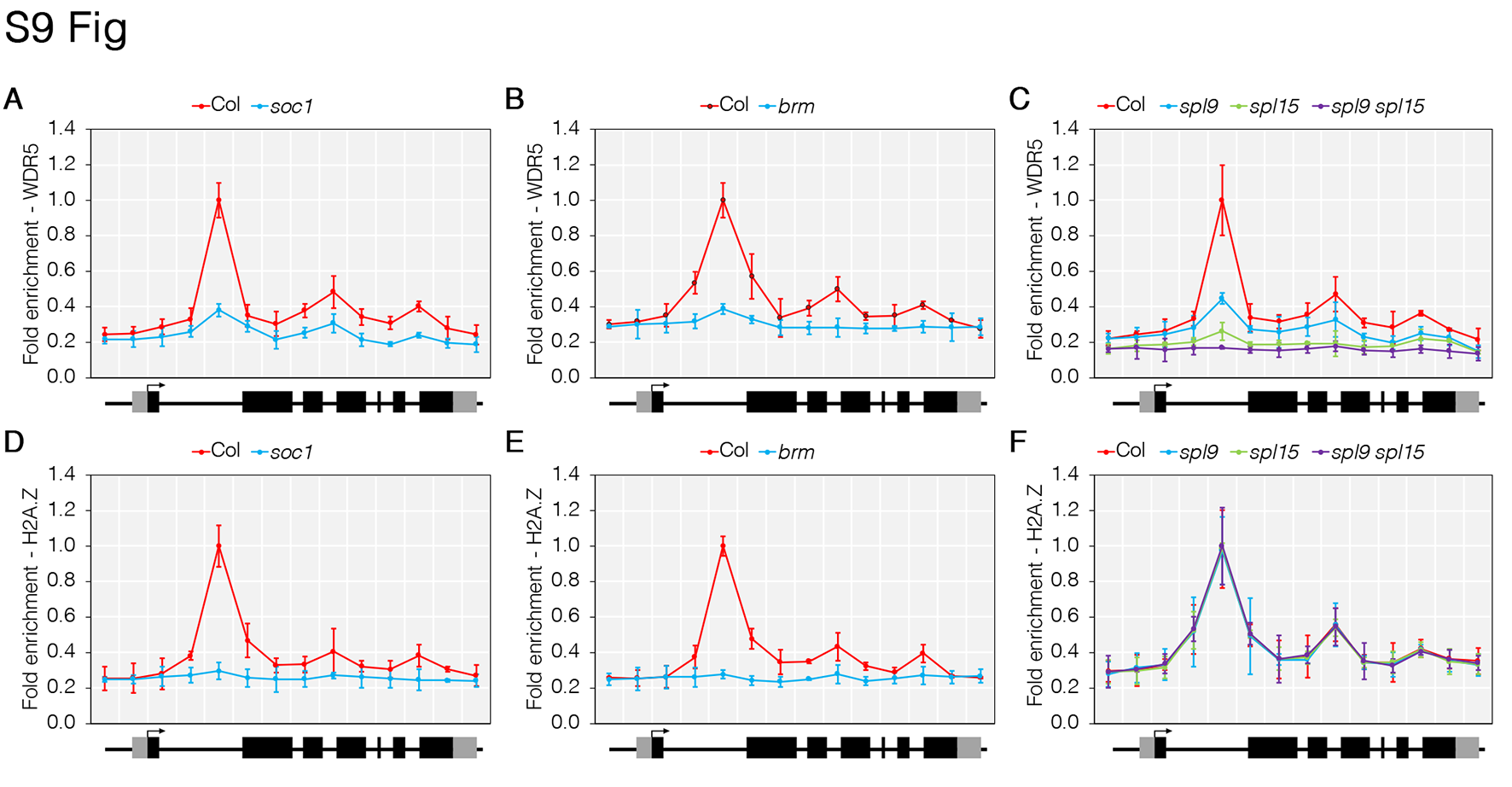

Supplement: S9 Fig — A to C) WDR5 levels at TFS1 depend on SOC1 (A), BRM (B) and SPL (C). D to F) H2A.Z incorporation at TFS1 depends on SOC1 (D) and BRM (E) but not on SPL (F). ChIP-PCR was performed on 15 LD grown plants that were harvested at ZT8 (A to F). (TIF) [file pgen.1008065.s009.tif]

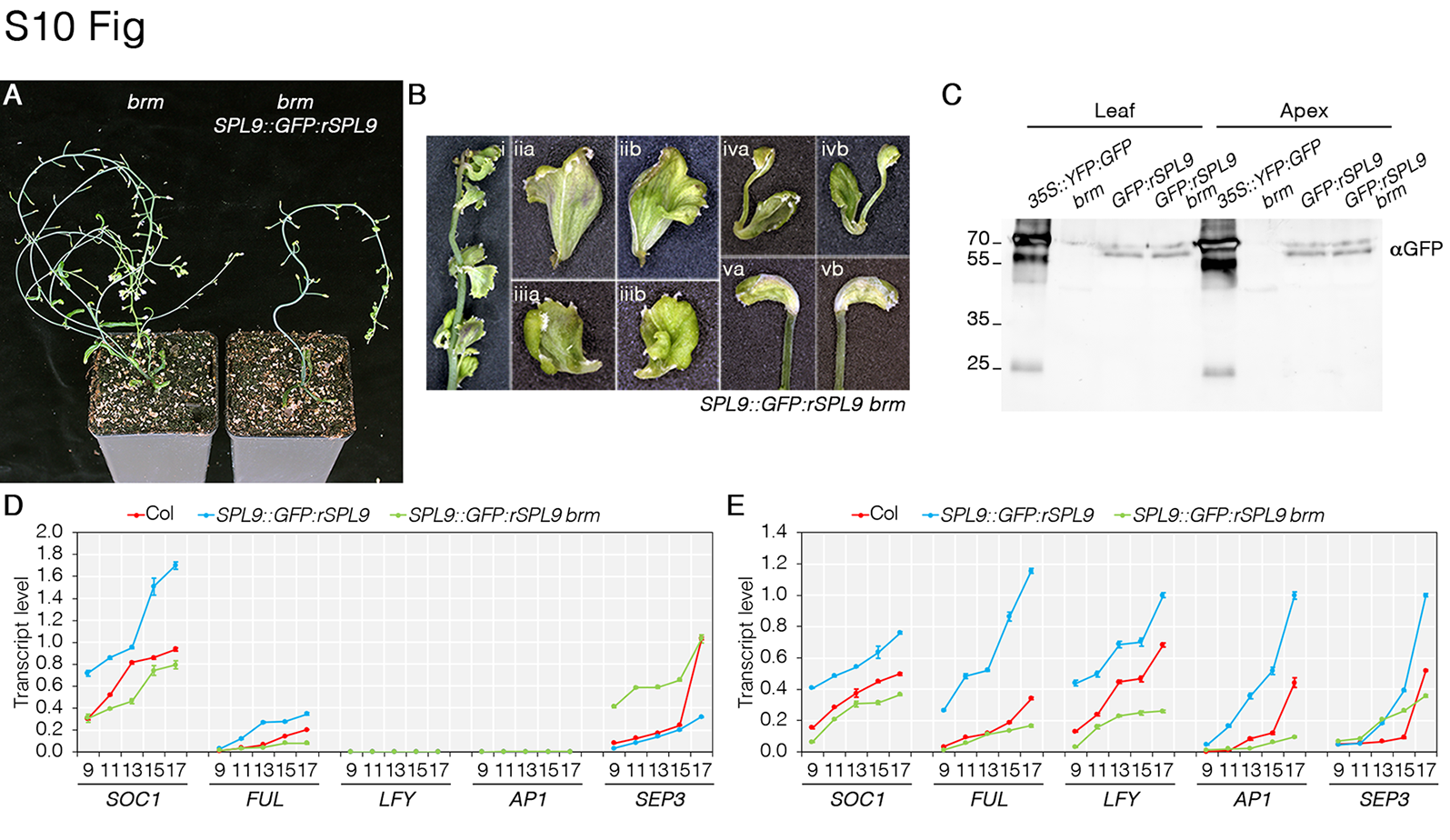

Supplement: S10 Fig — A and B) 5-weeks old long-day grown GFP:rSPL9 brm plants display altered plant architecture (A) and partial transformation of flowers into carpelloid structures (B). C) GFP Western analysis to detect GFP:SPL9 in Col and brm. Values on the left of the Western-blots indicated molecular weight (kDa). D and E) Comparative expression analysis of marker genes expressed in leaves (D) and apices (E) of Col, GFP:rSPL9 and GFP:rSPL9 brm plants. X-axis indicates number of long-days (LD) for which plants were grown prior to harvest. (TIF) [file pgen.1008065.s010.tif]
