# Supplementary material for: Floral regulators FLC and SOC1 directly regulate expression of the B3-type transcription factor TARGET OF FLC AND SVP 1 at the Arabidopsis shoot apex via antagonistic chromatin modifications
Source: PLoS Genet. 2019 Apr 4;15(4):e1008065. doi: 10.1371/journal.pgen.1008065 (PMC6467423; doi:10.1371/journal.pgen.1008065)
Supplement: S1 Table — (PDF) [file pgen.1008065.s011.pdf]

S1 Table		
**RESOURCES**	**SOURCE**	**IDENTIFIER**
**Experimental Models: Organisms/Strain**		
Arabidopsis: *brm-1*	Hurtado et al., 2006	N530046
Arabidopsis: *BRM::BRM:HA brm-1*	Efroni et al., 2013	N/A
Arabidopsis: *BRM::BRM:HA brm-1 ref6-1*	This study	N/A
Arabidopsis: *BRM::BRM:HA brm-1 soc1-2*	This study	N/A
Arabidopsis: *FRI-Col*	Mateos et al., 2015	N/A
Arabidopsis: *FRI-Col clf-2*	Hyun et al., 2013	N/A
Arabidopsis: *FRI-Col flc-3*	Mateos et al., 2015	N/A
Arabidopsis: *FRI-Col flc-3 svp-41*	Mateos et al., 2015	N/A
Arabidopsis: *FRI-Col flc-3 svp-41 SVP::SVP::GFP*	Mateos et al., 2015	N/A
Arabidopsis: *FRI-Col jmj14-2*	This study	N/A
Arabidopsis: *FRI-Col svp-41*	Mateos et al., 2015	N/A
Arabidopsis: *FRI-Col svp-41 SVP::SVP::GFP*	Mateos et al., 2015	N/A
Arabidopsis: *ga1*	Richter et al., 2010	N609115
Arabidopsis: *jmj14-2*	This study	N636058
Arabidopsis: *med18-2*	Hyun et al., 2016	N527178
Arabidopsis: *35S::miR156b*	Schwab et al., 2005	N/A
Arabidopsis: *ref6-1*	Lu et al., 2012	N501018
Arabidopsis: *35S::REF6:YFP-HA*	Lu et al., 2012	N/A
Arabidopsis: *REF6::REF6:HA ref6-1*	Lu et al., 2012	N/A
Arabidopsis: *REF6::REF6:HA ref6-1 soc1-2*	Hyun et al., 2016	N/A
Arabidopsis: *rga-28*	Hyun et al., 2016	N661007
Arabidopsis: *soc1-2*	Torti et al., 2012	N/A
Arabidopsis: *35S::SOC1:GR soc1-1*	Hyun et al., 2016	N/A
Arabidopsis: *35S::SOC1:myc(9x)*	Liu et al., 2008	N/A
Arabidopsis: *soc1-2 svp-41*	Torti et al., 2012	N/A
Arabidopsis: *soc1-2 TFS1::TFS1:9AV mCArGII*	This study	N/A
Arabidopsis: *spl9-1*	Schwarz et al., 2008	N/A
Arabidopsis: *spl15-1*	Schwarz et al., 2008	N/A
Arabidopsis: *spl9-1 spl15-1*	Schwarz et al., 2008	N/A
Arabidopsis: *SPL9::rSPL9::GR*	Wang et al., 2009	N/A
Arabidopsis: *SPL9::GFP:rSPL9*	Wang et al., 2009	N/A
Arabidopsis: *SPL9::GFP:rSPL9 brm-1*	This study	N/A
Arabidopsis: *svp-41*	Torti et al., 2012	N/A
Arabidopsis: *tfs1-1*	This study	N555624
Arabidopsis: *tfs1-2*	This study	N570274
Arabidopsis: *tfs1-1 TFS1::TFS1:9AV*	This study	N/A
Arabidopsis: *tfs1-1 TFS1::TFS1:9AV mCArGII*	This study	N/A
Arabidopsis: *tfs1-1 TFS1::TFS1:9AV mCArGII soc1-2*	This study	N/A

Arabidopsis: *tfs1-1 TFS1::TFS1:9AV mCArGI+II*	This study	N/A
Arabidopsis: *tfs1-1 TFS1::TFS1:9AV mGTACa1/a2*	This study	N/A
Arabidopsis: *35S::YFP-GFP*	Andrés et al., 2014	N/A
**Recombinant DNA**		
TFS1::TFS1:9xAla-Venus	This study	N/A
TFS1::TFS1:9xAla-Venus mCArG II	This study	N/A
TFS1::TFS1:9xAla-Venus mCArG I+II	This study	N/A
TFS1::TFS1:9xAla-Venus mGTACa1/a2	This study	N/A
