# Supplementary material for: Floral regulators FLC and SOC1 directly regulate expression of the B3-type transcription factor TARGET OF FLC AND SVP 1 at the Arabidopsis shoot apex via antagonistic chromatin modifications
Source: PLoS Genet. 2019 Apr 4;15(4):e1008065. doi: 10.1371/journal.pgen.1008065 (PMC6467423; doi:10.1371/journal.pgen.1008065)
Supplement: S2 Table — (PDF) [file pgen.1008065.s012.pdf]

S2 Table

Oligonucleotides

Genotyping primer	Sequence	AGI
brm-1 gLP	AATATACGCTTGCTGCATTGG	AT2G46020
brm-1 gRP	AGTTTATACCGTTGCATCCCC	AT2G46020
flc-3 gLP	TCGTCATGCGGTACACGTGGCAATC	AT5G10140
flc-3 gRP	TCGCCGGAGGAGAAGCTGTAGAGCT	AT5G10140
FRI-Col gLP	TGCTGGATTTGATAAGGATGAGTGGT	AT4G00650
FRI-Col gRP	TGGAACTTGATGTTGGTCGATGATG	AT4G00650
GFP gLP	CAATTCTTGTTGAATTAGATGGTG	AT5G60910
GFP gRP	GTGGACAGGTAATGGTTGTCTGGT	AT5G60910
jmj14-2 gLP	CCACACCAAGTTTTACATCCG	AT4G20400
jmj14-2 gRP	CCTGTCTATCGAGCTGTCCAG	AT4G20400
LB3	TAGCATCTGAATTTCATAACCAATCTCGATACAC	N/A
LBb1.3	ATTTTGCCGATTTCGGAAC	N/A
ref6-1 gLP	CAGACAGAGGAAATATGTTGTTGG	AT3G48430
ref6-1 gRP	TTCTTTTTCCTCCACATCTTTTTC	AT3G48430
rga-28 gLP	CCATCACCACCATTCTTTTTC	AT2G46020
rga-28 gRP	TGGACTAAACGAACACCGTTC	AT2G46020
soc1-2 WT gLP	TCTTCTTCTCCCTCCAGTAATGC	AT2G45660
soc1-2 WT gRP	TCTGAGTTTTGCCCCTCACCATATC	AT2G45660
soc1-2 MUT gLP	TTGGGTGATGGTTCACGTAGTGGG	AT2G45660
spl9-1 gLP	Schwarz et al., 2008	AT2G42200
spl9-1 gRP	Schwarz et al., 2008	AT2G42200
spl15-1 gLP	Schwarz et al., 2008	AT3G57920
spl15-1 gRP	Schwarz et al., 2008	AT3G57920
svp-41 gLP	GACCCACTAGTTATCAGCTCAG	AT2G22540
svp-41 gRP	AAGTTATGCCTCTCTAGGAC	AT2G22540
tfs1-1 gLP	TGTTTTCGTCTCCCATTTCAG	AT4G34400
tfs1-1 gRP	TCATCGTCATCAGAATCCTCC	AT4G34400
tfs1-2 gLP	CACTTCTGGTGAAGCAGCTTC	AT4G34400
tfs1-2 gRP	ACAGTCGATCCCATGATCAAG	AT4G34400
**qPCR primer**	**Sequence**	**AGI**
AP1 qLP	AAAACAGCATGCTTTCTAAACAGA	AT1G69120
AP1 qRP	GTGGCCTTGGTTCTGCTG	AT1G69120
FLC qLP	GCTACTTGAACTTGTGGATAGCAA	AT5G10140
FLC qRP	GGAGAGGGCAGTCTCAAGGT	AT5G10140
FUL qLP	TCACAACAATTCGCTTCTCAA	AT5G60910
FUL qRP	TTGGACTAATTGTCCTTCTTGCT	AT5G60910
LFY qLP	TTGATGCTCTCTCCCAAGAAG	AT5G61850
LFY qRP	TTGACCTGCGTCCCAGTAA	AT5G61850

SEP3 qLP	GAAAGCTGTACGAGTTTTGCAG	AT1G24260
SEP3 qRP	TTGAAGGCACATTGGGTTCT	AT1G24260
SOC1 qLP	AACAACTCGAAGCTTCTAAACGTAA	AT2G45660
SOC1 qRP	CCTCGATTGAGCATGTTCCT	AT2G45660
SPL9 qLP	GGCTGGTATCGAACAGAGGT	AT2G42200
SPL9 qRP	CAAATTCCGGAAGCTGATG	AT2G42200
SVP qLP	CCGGAAAACTGTTCGAGTTC	AT2G22540
SVP qRP	TGACTGCAAGTTATGCCTCTCT	AT2G22540
TFS1 qLP	GGTTGCAAATAAGGTTGCTAATC	AT4G34400
TFS1 qRP	TCTTGATCATGGGATCGACTT	AT4G34400
UBC21 qLP	TCCTCTTAACTGCGACTCAGG	AT5G25760
UBC21 qRP	GCGAGGCGTGTATACATTTG	AT5G25760
**Semi-qPCR primer**	**Sequence**	**AGI**
P1 LP	ACTCACTCTCTTCACACACAGATC	AT4G34400
P1 RP	ACTCTGAGCTGAAATGGGAGAC	AT4G34400
P2 LP	GATAATACTCTTAACGATGGAG	AT4G34400
P2 RP	AATCTCTTTACATGCGTCGTAGC	AT4G34400
P3 LP	AGACTATGTTAACTACATCGACAG	AT4G34400
P3 RP	TCTTGCATATGCGCATCCATCT	AT4G34400
UBC21 qLP	TCCTCTTAACTGCGACTCAGG	AT5G25760
UBC21 qRP	GCGAGGCGTGTATACATTTG	AT5G25760
**ChIP primer**	**Sequence**	**AGI**
ACT8 LP	CGTCTTCAGCTTCATCGGCCGTTGC	AT1G49240
ACT8 RP	ATCGAGATCCAACACAAGAACA	AT1G49240
TFS1 CArG I LP	AGCTGCTTCACCAGAAGTGAG	AT4G34400
TFS1 CArG I RP	ATGATCCAAGTCAAAGTTTTCA	AT4G34400
TFS1 CArG II LP	AGTTTTGTGGGTCCTATCGTCA	AT4G34400
TFS1 CArG II RP	ACCATGTCGACGTTGGATA	AT4G34400
TFS1 CArG III LP	TGTCAATGATTATTGTCAATGTG	AT4G34400
TFS1 CArG III RP	GTAGATATATCTCAACAATTCGG	AT4G34400
TFS1 GTAC-A LP	GAAATGGCCACGCAGAATACGAG	AT4G34400
TFS1 GTAC-A RP	GCTTGAGTCAAATCAACGTT	AT4G34400
TFS1 GTAC-B LP	ACAGAGGACTCATTCACTAAATA	AT4G34400
TFS1 GTAC-B RP	GAAGCAGCTTCCATTTAAGAGA	AT4G34400
TFS1 GTAC-C LP	CGATTAGAGTTGGTGATCGTT	AT4G34400
TFS1 GTAC-C RP	TCATTATCTCGCCGCGTCATTACT	AT4G34400
TFS1 GTAC-D LP	ACGTGTTCTTATCATTTCGACA	AT4G34400
TFS1 GTAC-D RP	TGTGATTAAGGGCGGCAGGCTCCT	AT4G34400
TFS1 GTAC-E LP	TGTCAATGTGACCATACTCTTCT	AT4G34400
TFS1 GTAC-E RP	GTAGATATATCTCAACAATTCGG	AT4G34400
TFS1 GTAC-F LP	CATACATAATCCAATAACAACA	AT4G34400

TFS1 GTAC-F RP	CATAACCAATTAAAACACATA	AT4G34400
TFS1 GTAC-G LP	ATATCTGAGTCCTATATCATTGA	AT4G34400
TFS1 GTAC-G RP	TCACCTAATATGAGACAAAATGAT	AT4G34400
TFS1 GTAC-H LP	ACCATAATGCAAGATTTTGGTA	AT4G34400
TFS1 GTAC-H RP	ATGACTAGATCCATCGTCTCA	AT4G34400
TFS1 Histone LP1	GTAGTGTGAAGATACATTCATG	AT4G34400
TFS1 Histone RP1	AGTAAATGTCTGTTAAACCGGGA	AT4G34400
TFS1 Histone LP2	GAGATCAAACCGTTTAAAAGAA	AT4G34400
TFS1 Histone RP2	TGAGTCAAATCAACGTTTAAGT	AT4G34400
TFS1 Histone LP3	ACTCACTCTCTTCACACACAGATC	AT4G34400
TFS1 Histone RP3	ACTCTGAGCTGAAATGGGAGAC	AT4G34400
TFS1 Histone LP4	ATCTGCATTTAGCTTCCTCTATAGA	AT4G34400
TFS1 Histone RP4	ACCAAAGGGTAGGAATAAGAGCA	AT4G34400
TFS1 Histone LP5	AGTATAAAGTAGTTTTGCTCAGA	AT4G34400
TFS1 Histone RP5	ACCAGACAAAATCAAAGCATCCA	AT4G34400
TFS1 Histone LP6	ATGACTCTCTCCGACTTACCTTA	AT4G34400
TFS1 Histone RP6	GATCACCTGCTCATTTCACAGAA	AT4G34400
TFS1 Histone LP7	GATAATACTCTTAACGATGGAG	AT4G34400
TFS1 Histone RP7	AATCTCTTTACATGCGTCGTAGC	AT4G34400
TFS1 Histone LP8	TGCAGGAAAGTCAAAAGTGGAAG	AT4G34400
TFS1 Histone RP8	ATCAGTGTCTGTCTCTGTTTCTTC	AT4G34400
TFS1 Histone LP9	AGAAAGAACAAGTTGTGGAGTC	AT4G34400
TFS1 Histone RP9	ATTCTCTTCAATATCCAATTGAC	AT4G34400
TFS1 Histone LP10	ATCTTAATGCAACATTGCTTTGTC	AT4G34400
TFS1 Histone RP10	ATTACATAATGAAGTGATGGAAC	AT4G34400
TFS1 Histone LP11	AGAATCCTGAGAGATACCTCGA	AT4G34400
TFS1 Histone RP11	ACAAGAAGTTGCAGTTCTTACCA	AT4G34400
TFS1 Histone LP12	AGACTATGTTAACTACATCGACAG	AT4G34400
TFS1 Histone RP12	TCTTGCATATGCGCATCCATCT	AT4G34400
TFS1 MNase 2.1 LP1	AGACTATGTTAACTACATCGACAG	AT4G34400
TFS1 MNase 2.1 RP1	TCTTGCATATGCGCATCCATCTT	AT4G34400
TFS1 MNase 2.2 LP1	TGGAAGGATCAGCGTGTTTGT	AT4G34400
TFS1 MNase 2.2 RP1	AGTTCCCTTGCGCAAGAGCTCA	AT4G34400
TFS1 MNase 2.3 LP2	ACGCAATCAAACTCCATGTCATCC	AT4G34400
TFS1 MNase 2.3 RP2	AACAAGATGGAAATCAGAGAGATA	AT4G34400
TFS1 MNase 2.4 LP3	ACTTATGTGTTTCATTATTGTCTAC	AT4G34400
TFS1 MNase 2.4 RP3	GCTCAAAGATTAGATATTGTATTCAG	AT4G34400
TFS1 MNase 2.5 LP4	TCTTTGAGCTAAAGTTTAACTCT	AT4G34400
TFS1 MNase 2.5 RP4	ACAGTTTTCGACCTTTCTTTGA	AT4G34400
TFS1 MNase 2.6 LP5	TCGAAAACTGTATATTTGTCTTAGC	AT4G34400
TFS1 MNase 2.6 RP5	TCCGTGGAAACCAGTATACCTAGAT	AT4G34400

TFS1 MNase 2.7 LP6	TCAAAGTAGAAGAAACAGAGGACTC	AT4G34400
TFS1 MNase 2.7 RP6	ACGAGGATGAAGAAGGAAGGTACA	AT4G34400
TFS1 MNase 2.8 LP7	ATGTACCTTCCTTCTTCATCCTCG	AT4G34400
TFS1 MNase 2.8 RP7	CAAAGCTTTCAAGGTCATTTCTCAC	AT4G34400
TFS1 MNase 2.9 LP8	ATGACCTTGAAAGCTTTGTGACA	AT4G34400
TFS1 MNase 2.9 RP8	TACTCTGATAATGGAAGTTACT	AT4G34400
TFS1 MNase 3.0 LP9	ATACTAACCAACATAAAGTTTGA	AT4G34400
TFS1 MNase 3.0 RP9	ATATCCAACGTCGACATGGTAAAC	AT4G34400
TFS1 MNase 3.1 LP10	ATTCCAGTTTTGTGGGTCCTATC	AT4G34400
TFS1 MNase 3.1 RP10	AGCTTGCTCTGTTTTGTAAAGATGA	AT4G34400
TFS1 MNase 3.2 LP11	TCTGTTTCGAGTCCAAAAATAGT	AT4G34400
TFS1 MNase 3.2 RP11	ATAACAAATGATCCAAATCAGAG	AT4G34400
TFS1 MNase 3.3 LP12	ATCATTTGTTATTTTCTAGTTACA	AT4G34400
TFS1 MNase 3.3 RP12	AGGACCCACAAAAATGGAAGTATG	AT4G34400
**3C primer**	**Sequence**	**AGI**
TFS1 3C LP-A	ACTCAAGCGTCAAGGTAAGGTAC	AT4G34400
TFS1 3C LP-B	TACTTATCTATTGAGTGTTTGGCT	AT4G34400
TFS1 3C LP-C	ATGGAACCAGAGGCGGCAAAGAAG	AT4G34400
TFS1 3C LP-D	ATCGACAGGTTCGGGAAACTAAGTGC	AT4G34400
TFS1 3C LP-E	ATTCTGAAGAATCTAGGTATACTG	AT4G34400
TFS1 3C LP-F	TCAGAGTAAAATATACTAACCAAC	AT4G34400
TFS1 3C LP-G	ATCATTATCATAGTAAACATACAAC	AT4G34400
TFS1 3C LP-H	ATGGTGTAGACCTTAACCAGCTC	AT4G34400
TFS1 anchor LP-B	ATGGACTTAACACTGGATATG	AT4G34400
TFS1 anchor RP-B	TCAGCATTTAGAAGCAGAACAAATG	AT4G34400
TFS1 anchor LP-C	ACACAGCCACTGCCTCTTTTGTCA	AT4G34400
TFS1 anchor RP-C	TCTGGTTCCATAGACAAAGCAATGT	AT4G34400
TFS1 anchor LP-G	ATAGTTTACCATGTCGACGTTGGA	AT4G34400
TFS1 anchor RP-G	ACAGAGTAGCTTGCTCTGTTTTGTA	AT4G34400
***in-situ* hybridization**		
TFS1 558V241	ATTAACCCTCACTAAAGGGACAGCTCCCTCCACG TTTCTT	AT4G34400
TFS1 558V242	TAATACGACTCACTATAGGGTATTGTTGGCTTGG CCACCT	AT4G34400
TFS1::TFS1:9Ala-Venus		
TFS1 RM1	GGGGACAAGTTTGTACAAAAAAGCAGGCTTC TATTTTTTCCTAAAATTCTTTCTTTCTTTGTGGA AATTACA	AT4G34400
TFS1 RM2	GGGGACCACTTTGTACAAGAAAGCTGGGTCC TAACCCGACTCTCGATTCCTTCTTCTG	AT4G34400
TFS1 RM3	TGTCATCCGCGGTAAGGATCTGATGGCTGCG GCTGCTG	AT4G34400

TFS1 RM4	CAGCAGCCGCAGCCATCAGATCCTTACCGCG GATGACA	AT4G34400
TFS1 RM5	CGGCATGGACGAGCTGTACAAGTAAACGCTA GCAGTATGAGTAGAGGAAGAGATTTG	AT4G34400
TFS1 RM6	CAAATCTCTTCCTCTACTCATACTGCTAGCGT TTACTTGTACAGCTCGTCCATGCCG	AT4G34400
TFS1 RM7	CTGATAATGGAAGTTATTATTTTTTGACTAAAA ACAGAGTAGCTACTCTGTTTTATATCACAAAA TTCACAATTATTATGTCACA	AT4G34400
TFS1 RM8	AGCTACTCTGTTTTTAGTCAAAAAATAATAACT TCCATTATCAGAGTAAAATATACTAACCAACA TAAAGTTTGATTATTTTGAAAACTTTG	AT4G34400
TFS1 RM9	TGATTGTTATTATTTTTTGACTCGAAACAGAGT AGCTTGCTCTGTTTTGTAAAGATGAAAGCAAA C	AT4G34400
TFS1 RM10	GCTACTCTGTTTCGAGTCAAAAAATAATAACAATC ATTATCATAGTAAACATACAACTAAACCAACCCTA AAGTTTTATTATTT	AT4G34400
TFS1 RM11	CAGAGAAAGGAGTTAAAATCCTCAGACCCTCGTA TTCTGCGTGGCCATTTC	AT4G34400
TFS1 RM12	GGGTCTGAGGATTTTAACTCCTTTCTCTGCAAAA ACTTAAACGTTGATTTGACTCAAGC	AT4G34400
TFS1 RM13	TTGTATGAAGAGAGATTAACTTACCTTGACGCTT GAGTCAAATCAACGTTTAAGTTTTTGCAGAG	AT4G34400
TFS1 RM14	AAGCGTCAAGGTAAGTTAATCTCTCTTCATACAA CATTTTAGCTTTACTTTTTCTCTTTACTCTTCTCTC TCTCTTT	AT4G34400
TFS1 RM15	CTTCACCAGAAGTGAGGAGAAACAAA	AT4G34400
TFS1 RM16	ATTCTTTCTTTGGATATGCTCCACTTTTTG	AT4G34400
TFS1 RM17	TCAACCAAAAGCGTAGGGACAGT	AT4G34400
