# Supplementary material for: Floral regulators FLC and SOC1 directly regulate expression of the B3-type transcription factor TARGET OF FLC AND SVP 1 at the Arabidopsis shoot apex via antagonistic chromatin modifications
Source: PLoS Genet. 2019 Apr 4;15(4):e1008065. doi: 10.1371/journal.pgen.1008065 (PMC6467423; doi:10.1371/journal.pgen.1008065)
Supplement: S3 Table — (PDF) [file pgen.1008065.s013.pdf]

S3 Table		
**RESOURCES**	**SOURCE**	**IDENTIFIER**
**Antibodies**		
Mouse monoclonal anti-FLAG (clone M2)	Sigma Aldrich	F1804-200UG
Rabbit polyclonal anti-FLC	Mateos et al., 2017	N/A
Rabbit polyclonal anti-GFP (ChIP grade)	Abcam	ab290
Rabbit polyclonal anti-GR	Thermo Fisher Scientific	PA1-516
Rabbit polyclonal anti-H2A.Z (ChIP grade)	Abcam	ab4174
Rabbit polyclonal anti-H3 (ChIP grade)	Abcam	ab1791
Rabbit polyclonal anti-HA tag (ChIP grade)	Abcam	ab9110
Rabbit polyclonal anti-K4 trimethyl-Histone H3	Abcam	ab8580
Rabbit polyclonal anti-K27 trimethyl-Histone H3	Merck	#07-449
Rabbit polyclonal anti-LHP1	Agrisera	AS13 2675
Rabbit polyclonal anti-MED18	Santa Cruz Biotech.	sc-367 125
Goat polyclonal anti-Myc tag (ChIP grade)	Abcam	ab9132
Rabbit polyclonal anti-RGA	Agrisera	AS11 1630
Mouse monoclonal anti-RNAPII [8WG16]	Abcam	ab817
Rabbit polyclonal anti-SOC1	Hyun et al., 2016	N/A
Rabbit polyclonal anti-SVP	This study	N/A
Rabbit polyclonal anti-WDR5	Abcam	ab75439
**Chemicals**		
Buffer NTB	Macherey-Nagel	#740.595.150
CutSmart Buffer, 5mL	New England Biolabs	B7204S
Di(N-succinimidyl) glutarate (DSG)	Synchem	bc366
Dynabeads M-270 Streptavidin	Thermo Fisher Scientific	#65305
GlycoBlue Coprecipitant (15mg/mL)	Thermo Fisher Scientific	AM9516
GoTaq qPCR Master Mix	Promega	A6002
iQ SYBR Green Supermix	BioRad	#170-8882
Micrococcal nuclease	Thermo Fisher Scientific	EN0181
Miracloth	Calbiochem	475855-1R
Percoll	GE Healthcare	17-0891-02
Phenol:Chloroform:Isoamylalcohol (25:24:1)	Sigma Aldrich	P2069-400ML
PIPES	Sigma Aldrich	P6757-500G
Protease Inhibitor Cocktail	Sigma Aldrich	P9599-5ML
Protein A Sepharose Fast Flow	GE Healthcare	#17-1279-01
RNAseOUT Recombinant Ribonuclease Inhibitor	Thermo Fisher Scientific	#10777019
Sau3AI, 1.000Units	New England Biolabs	R0169L
SUPERase INTM RNase Inhibitor, 2.500U	Thermo Fisher Scientific	AM2694
SuperScript II Reverse Transcriptase, 10.000U	Thermo Fisher Scientific	#18064014
T4 DNA Ligase (HC), 500U	Promega	M1794
T4 DNA Ligase Buffer	Promega	C1263

Vector-Fusion-Aid GFP Kit	Axxora	VC-MB-0732-KI01
**Critical Commercial Assays**		
NucleoSpin Gel & PCR clean up	Macherey-Nagel	#740.609.250
NucleoSpin RNA Plant (250)	Macherey-Nagel	#740.949.250
SuperSignal West Dura Extended Duration	Thermo Fisher Scientific	#34076
