# Supplementary material for: Floral regulators FLC and SOC1 directly regulate expression of the B3-type transcription factor TARGET OF FLC AND SVP 1 at the Arabidopsis shoot apex via antagonistic chromatin modifications
Source: PLoS Genet. 2019 Apr 4;15(4):e1008065. doi: 10.1371/journal.pgen.1008065 (PMC6467423; doi:10.1371/journal.pgen.1008065)
Supplement: S4 Table — (PDF) [file pgen.1008065.s014.pdf]

S4 Table

Amino acid sequences of epitopes for antibody production

Antibody	Sequence	Epitope position in protein
SVP	LGMQICNNVHAHGGAESENAAVYEEGQSSESITNAGNSTG APVDSESSDTSLRLGLPYGG	181aa to 240aa
