# Supplementary material for: Floral regulators FLC and SOC1 directly regulate expression of the B3-type transcription factor TARGET OF FLC AND SVP 1 at the Arabidopsis shoot apex via antagonistic chromatin modifications
Source: PLoS Genet. 2019 Apr 4;15(4):e1008065. doi: 10.1371/journal.pgen.1008065 (PMC6467423; doi:10.1371/journal.pgen.1008065)
Supplement: S5 Table — (PDF) [file pgen.1008065.s015.pdf]

S5 Table

Conditions (LD)	Leaf numbers		Days	Number of analysed plants
	Rosette	Cauline		
Col	14.96±1.10	3.36±0.70	24.88±1.05	25
tfs1-1	17.00±1.23	4.72±0.74	32.36±1.15	25
TFS1::TFS1:9xAV tfs1-1 #6	14.92±0.76	3.44±0.65	24.76±0.66	25
TFS1::TFS1:9xAV tfs1-1 #12	14.76±0.93	3.32±0.56	24.48±0.92	25
TFS1::TFS1:9xAV tfs1-1 #25	14.72±0.98	3.32±0.63	24.44±0.87	25
TFS1::TFS1:9xAV tfs1-1 #33	14.32±1.15	3.16±0.37	22.88±1.13	25

Conditions	Leaf numbers		Days	Number of analysed plants
	Rosette	Cauline		
Col	15.08±1.00	3.40±0.50	24.96±0.84	25
tfs1-1	16.96±1.06	4.76±0.72	32.20±1.35	25
TFS1::TFS1:9xAV mCArGII tfs1-1 #4	14.92±0.70	3.23±0.44	24.60±0.70	25
TFS1::TFS1:9xAV mCArGII tfs1-1 #5	14.56±0.77	3.20±0.41	23.48±0.82	25
TFS1::TFS1:9xAV mCArGII tfs1-1 #7	14.36±0.70	3.16±0.37	23.24±0.78	25
TFS1::TFS1:9xAV mCArGII tfs1-1 #11	14.00±0.49	3.08±0.49	22.84±0.94	25

Conditions	Leaf numbers		Days	Number of analysed plants
	Rosette	Cauline		
Col	15.08±0.81	3.40±0.65	25.16±0.69	25
tfs1-1	17.20±0.65	4.72±0.68	32.48±0.82	25
TFS1::TFS1:9xAV mCArGI+II tfs1-1 #3	17.16±0.90	4.88±0.78	32.52±0.96	25
TFS1::TFS1:9xAV mCArGI+II tfs1-1 #5	16.80±0.58	4.60±0.65	31.32±0.56	25
TFS1::TFS1:9xAV mCArGI+II tfs1-1 #8	16.68±0.96	4.52±0.65	30.56±1.08	25
TFS1::TFS1:9xAV mCArGI+II tfs1-1 #12	16.60±0.82	4.48±0.59	30.24±0.97	25

Conditions	Leaf numbers		Days	Number of analysed plants
	Rosette	Cauline		
Col	15.04±0.89	3.44±0.51	24.84±0.90	25
soc1-2	31.72±1.31	4.76±0.66	41.76±1.20	25
TFS1::TFS1:9xAV mCArGII soc1-2 #4	30.08±2.02	4.12±0.60	40.68±1.46	25
TFS1::TFS1:9xAV mCArGII soc1-2 #5	29.80±1.73	4.04±0.61	40.32±1.25	25
TFS1::TFS1:9xAV mCArGII soc1-2 #7	29.48±2.04	3.96±0.54	39.68±1.35	25
TFS1::TFS1:9xAV mCArGII soc1-2 #11	29.28±1.97	3.96±0.61	39.68±1.28	25

Conditions	Leaf numbers		Days	Number of analysed plants
(LD)	Rosette	Cauline		
Col	14.16±1.14	3.24±0.72	24.24±1.62	25
jmj14-2	11.12±1.13	2.92±0.57	21.08±1.73	25
FRI-Sf2	55.88±4.71	14.68±1.41	44.88±3.36	25
FRI-Sf2 jmj14-2	18.56±1.89	4.08±1.22	28.36±2.86	25
FRI-Sf2 flc-3 svp-41	6.92±0.91	2.48±0.65	19.64±1.38	25
